# Assessing inequalities in paediatric emergency department admissions in the Northwest of England: a retrospective cohort study

**DOI:** 10.1136/bmjph-2025-002916

**Published:** 2026-06-19

**Authors:** Courtney Franklin, Bernie Carter, David Taylor-Robinson, Enitan Carrol

**Affiliations:** 1 University of Liverpool Faculty of Health and Life Sciences, Liverpool, UK; 2 Edge Hill University, Ormskirk, UK; 3 Public Health, Policy and Systems, University of Liverpool, Liverpool, UK; 4 Clinical Infection, Microbiology and Immunology, University of Liverpool, Liverpool, UK

**Keywords:** Community Health, Confounding Factors, Epidemiologic, Health Services Accessibility, Public Health, Sociodemographic Factors

## Abstract

**Introduction:**

Prior to the UK’s first COVID-19 related lockdown (2020), the number of paediatric emergency department (ED) attendances and admissions from ED had increased. Predictors of admission to a paediatric ED include patient characteristics and reasons for admission. With evidence of a social gradient in paediatric emergency hospital attendances and admissions, the rise in admissions could also reflect socio-economic conditions (SECs).

**Methods:**

To investigate the effect of SECs on admission to hospital from ED, we analysed data on all children presenting to the ED at Alder Hey Children’s Hospital in Northwest England, between 2016 and 2019 (206 918 attendances). Logistic regression models and generalised additive models assessed the association between SECs (using Index of Multiple Deprivation (IMD)) and risk of admission from the ED, conditional on ED attendance. We further explored the social patterning of other important factors, such as referral source, diagnosis and triage category. Logistic regression models additionally adjusted for these important variables to investigate if these factors helped to explain the association between deprivation and risk of admission from the ED.

**Results:**

Admission occurred in approximately 16% of attendances among children living in areas of lower SECs compared with 24% among their most affluent counterparts. In unadjusted analyses, increasing deprivation was associated with lower odds of admission conditional on ED attendance (OR per one-unit increase in IMD 0.996, 95% CI 0.995 to 0.996). After adjustment for demographic, case-mix and clinical presentation variables, the association reversed direction (OR 1.002, 95% CI 1.000 to 1.003). However, in generalised estimating equation sensitivity analyses accounting for repeated attendances, the fully adjusted association attenuated towards the null.

**Conclusions:**

Associations between SECs and admission risk conditional on ED attendance were sensitive to adjustment for case-mix and clinical presentation variables and to modelling strategy. Results reflect differences in clinical decision-making, risk tolerance or unmeasured social factors influencing admission decisions.

**WHAT IS ALREADY KNOWN ON THIS TOPIC**

- Children remain frequent users of emergency healthcare, with the most disadvantaged children experiencing more emergency admissions than their more affluent peers.
- Case mix and care pathway characteristics such as presenting concern, triage category, referral source and weekday attendance impact risk of admission to hospital from paediatric emergency department (ED).

**WHAT THIS STUDY ADDS**

- Children living in areas of lower socio-economic conditions (SECs) were likely to have fewer investigations performed, be triaged as ‘Green’ or ‘GP appropriate’, attend the ED out of hours, self-referrals, have shorter inpatient stay and have diagnoses of infectious disease and respiratory conditions compared with their most affluent counterparts.
- After adjustment of case-mix and clinical presentation variables and conditional on ED attendance, children experiencing lower SECs were more likely to be admitted from the ED than their most affluent peers.
- Our results may reflect differences in clinical decision-making, risk tolerance or unmeasured social factors influencing admission decisions, potentially reducing some impacts of social disadvantage such as allowing clinicians to identify vulnerable children who may benefit from more comprehensive or earlier interventions.

**HOW THIS STUDY MIGHT AFFECT RESEARCH, PRACTICE OR POLICY**

- Interventions aiming to reduce unnecessary emergency hospital use, especially for children with low disease acuity, should focus on communities of higher deprivation to ensure equitable access to services and safety netting advice.
- A balanced approach to consideration of SECs in clinical decision-making can help reduce health disparities while avoiding unintended consequences such as perpetuation of stereotypes.

## Introduction

 Children remain frequent users of emergency healthcare, yet many presenting problems to the emergency departments (EDs) are self-limiting and may not require ED attendance.^1^ Prior to the UK’s first COVID-19 related lockdown (March 2020), increasing trends in ED attendance and admissions were observed for children and young people^2 3^; at the same time, paediatric general practice consultations fell.^3^ There has also been an increase in short stay (typically less than 48–72 hours) unplanned and short stay urgent admissions, despite demographics of these children remaining unchanged.^4^,^8^ These findings may reflect barriers to primary care access as well as decreased parental capacity or willingness to manage self-limiting childhood illnesses at home rather than increased burden of disease necessitating emergency care.^9^

These trends may also reflect factors such as socio-economic conditions (SECs). A social gradient in emergency admissions has been established, whereby socio-economically disadvantaged (lower SECs) people are more likely to present to the ED than those who are economically advantaged.^10^,^16^ Between 2007 and 2017, divergent patterns of healthcare use along a social gradient were found among children in England; children living in more socio-economically disadvantaged areas made greater use of emergency services and received less scheduled care compared with their most affluent counterparts.^217^,^20^ A previous study in England found a positive association between socio-economically disadvantage and ED attendance rates, where increased disadvantage was associated with increased attendance. However, no association was found with conversion probability (the probability of being admitted after having attended an ED department).^21^

Many studies have assessed how case mix and care pathway characteristics impact risk of admission to hospital for children from ED. Some of the predictors include presenting concern,^22 23^ triage category,^22^,^26^ referral source,^22^,^2427^ history of previous admission,^22^ triage vital signs (such as heart rate, respiratory rate and temperature),^23 27^ age,^23 27 28^ season of attendance^25^ and weekday attendance.^22^ It is important to understand drivers of admissions to allocate resources more effectively and ensure more equitable care, where disadvantaged populations are not disproportionately impacted by worse health outcomes.

With evidence of a social gradient in paediatric admission rates in England,^28^ we used data from all ED attendances in a tertiary children’s hospital setting in Liverpool, across a 4-year period in an area of high socio-economic disadvantage, to investigate the effect of SECs on ED admission, conditional on attendance and explore the distribution of SECs on important outcomes from all paediatric ED attendances.

## Materials and methods

### Data source

We undertook a retrospective cohort study using data collected from Alder Hey Children’s NHS Foundation Trust, a tertiary children’s hospital situated in Liverpool, UK. Liverpool city has a higher than national average population of children from the most socio-economically disadvantaged backgrounds, with approximately 28% of children in low income families^29^ and 24 000 children living in poverty in Liverpool.^30^ We studied all data in Alder Hey from 2016 to 2019, collected as part of a larger study, the PERFORM study (Personalised Risk assessment in Febrile illness to Optimise Real-life Management across the European Union).^20 31 32^ Consecutive febrile children, defined as a temperature above 38°C at presentation, or below 38°C with a history of fever up to 3 days previous, were prospectively identified.

### Study population

We included all children (aged ≤18 years) presenting to the ED at Alder Hey Children’s NHS Foundation Trust, a large paediatric specialist care provider in the Northwest of England, Liverpool, between 1 January 2016 and final discharge on 31 December 2019. Any child listed was included regardless of any diagnosis, provided they had available postcode data to link to Index of Multiple Deprivation (IMD) scores. Patients were only subsequently excluded if data regarding key components of their stay, including admission and investigations/medical interventions provided, or healthcare personnel seen, were missing or incomplete.

The original dataset comprised 331 591 ED attendances between 2015 and 2020. Attendances without a recorded postcode were excluded because postcode was required to derive the IMD score. Data from 2020 were excluded due to potential disruptions in ED attendance patterns during the COVID-19 pandemic, and data from 2015 were removed to ensure a consistent study period. All paediatric attendances were eligible regardless of diagnosis, provided a postcode was available for linkage to IMD. After these exclusions, 240 653 attendances remained. Restricting the dataset to observations with complete data for all variables included in the analyses resulted in a final analytical sample of 206 918 attendances (86%). Online supplemental figure S1 provides a flow chart to represent this.

### Outcomes

^20 31 32^ The primary outcome of interest was hospital admission from the ED, conditional on attendance. While our study focuses on the outcome of admission, we also derived a factor variable indicating inpatient stay to assess any further social patterning^31^: less than 1 day (1); between 1 and 3 days (2); between 4 and 7 days (3); 8 days and over (4). This method has been used elsewhere.^31^

### Exposure

The primary exposure variable was the IMD score, a measure of socio-economically disadvantaged area of residence. This is the most widely used index in the English Indices of Deprivation organised within the seven domains of income, employment, education, health, crime, barriers to housing and services and living environment.^33^ Postcode data were attributed to each patient’s LSOA (Lower Super Output Area) of residence. We obtained IMD data for each LSOA, including score and decile using 2019 Census data^34^ and used the continuous IMD variable as a fixed measure of SECs. The continuous IMD score ranges from scores between approximately 0 and 80.

### Covariate

Regression analyses were adjusted for age, gender, year and season to control for the potential confounding effects on the relationship between socio-economic disadvantage and emergency hospital admissions. Additional important covariates describing case-mix and clinical presentation variables were also explored, including out of hours attendance, number of investigations performed and diagnosis. Similar predictors of paediatric emergency health service use have been used elsewhere.^20 22 25^

### Conceptual framework

The logic model (online supplemental figure S2) describes the hypothetical pathway between socio-economic disadvantage and hospital admission and subsequent length of stay, and the potential effects of the covariates we had data for. Variables describing case-mix and clinical presentation at attendance (including triage category, referral source, diagnosis, out-of-hours attendance and number of investigations) were considered potential explanatory factors that may account for differences in admission risk across socio-economic groups. These variables were added sequentially to regression models to examine how adjustment for clinical characteristics influenced the association between deprivation and admission. This approach is intended to explore potential explanatory pathways rather than to formally estimate mediation effects. We assumed that low SECs may contribute to increased severity of illness at presentation, which may subsequently increase the likelihood of hospital admission.^35^ Using this logic model, our sequential model approach helps to test to what extent, if any, case-mix and clinical presentation variables explain the relationship between socio-economic disadvantage and paediatric hospital admission, conditional on attendance.

A full description of the measures and data sources can be found in the supplementary file (online supplemental table S1).

### Statistical analyses

First, we described the distribution of socio-economic disadvantage from all admissions within the study population compared with the rest of England, using data retrieved from Public Health England^36^ to help illustrate the selection in our study population, where Liverpool is a more socio-economically disadvantaged area than the national average, and attendance patterns should be interpreted in this context. We then presented summary statistics of the prevalence of admission and other key factors within the study population, stratified by English IMD deciles (common practice for descriptive summaries).

We explored the univariate relationships between the log-odds of admission, conditional on attendance, and IMD score using a Generalised Additive Model (GAM), and used IMD score as a continuous measure of scores between approximately 0 and 80 for all formal analysis, to prevent loss of information resulting from reducing IMD to a categorical variable.

We used binary logistic regression models to estimate the association between IMD score and odds of hospital admission conditional on ED attendance. IMD was modelled as a continuous variable to retain information across the full deprivation distribution. While ORs are presented, these can be transformed to the Relative Index of Inequality for further interpretability, which represent the relative odds of admission comparing the most advantaged group compared with the least advantaged group across the SEC hierarchy, while using information from the full distribution.^37^

Because some children contributed multiple ED attendances during the study period, observations were not fully independent. To account for within-child correlation, we estimated logistic regression models with cluster-robust SEs using patient identifier as the clustering unit. Robust variance (sandwich) estimators adjust SEs to account for correlation within clusters without altering coefficient estimates. This approach reduces the risk of underestimated SEs and inflated type I error when repeated observations occur within individuals.

To explore if the relationship between admission and socio-economic disadvantage, we fitted three nested models informed by our logic model (online supplemental figure S1): an unadjusted model to quantify the association between odds of admission and socio-economic disadvantage (Model 1); a model adjusted for baseline demographic variables (age, gender, season and arrival year) (Model 2); a model additionally adjusted for case-mix and clinical presentation variables, to explore if the initial association is affected by these (Model 3). If these case-mix and clinical presentation variables that lie on the pathway between socio-economic circumstances and admission, Model 3 will help to illustrate the mechanism behind the observed relationship in Model 2. We took any substantial change in association after adjustment for these variables to indicate that differences in clinical presentation and care pathways may explain observed socio-economic differences in admission.^38 39^ Regression-based approaches that examine attenuation after adjustment for variables that may lie on the causal pathway have been widely used to explore potential explanatory pathways. However, this approach does not in itself constitute formal mediation analysis and relies on strong assumptions. We did not conduct formal mediation analysis; therefore, findings should be interpreted as associations rather than evidence of causal mediation. A predetermined significance level was set at 5% (p<0.05), decided on through common convention.^40^

To further explore the relationship between socio-economic disadvantage and risk factors for admission, we conducted univariate analyses, including tests of association and visualisations of the unadjusted distribution of key variables by IMD decile. For this analysis, we use IMD deciles for clearer visual representation and to easily compare the most and least disadvantaged groups in the study population.

### Robustness tests

The primary analyses used logistic regression with cluster-robust SEs because the study aimed to estimate attendance-level associations while accounting for within-child correlation in inference. However, we additionally fitted generalised estimating equation (GEE) models as an extension of logistic regression analyses to estimate population-averaged associations accounting for repeated attendances. GEE models were specified with patient identifier as the clustering unit, producing population-averaged estimates. Results from these models were compared with the primary logistic regression models with cluster-robust SEs.

### Patient and public involvement

Given the nature of this study, there were no direct interactions with patients or the public. However, this study is part of a wider PhD project that involved qualitative work with parents and practitioners^41^ as well as a public advisor which subsequently informed this analysis.

## Results

### Descriptive results

**Figure 1:**
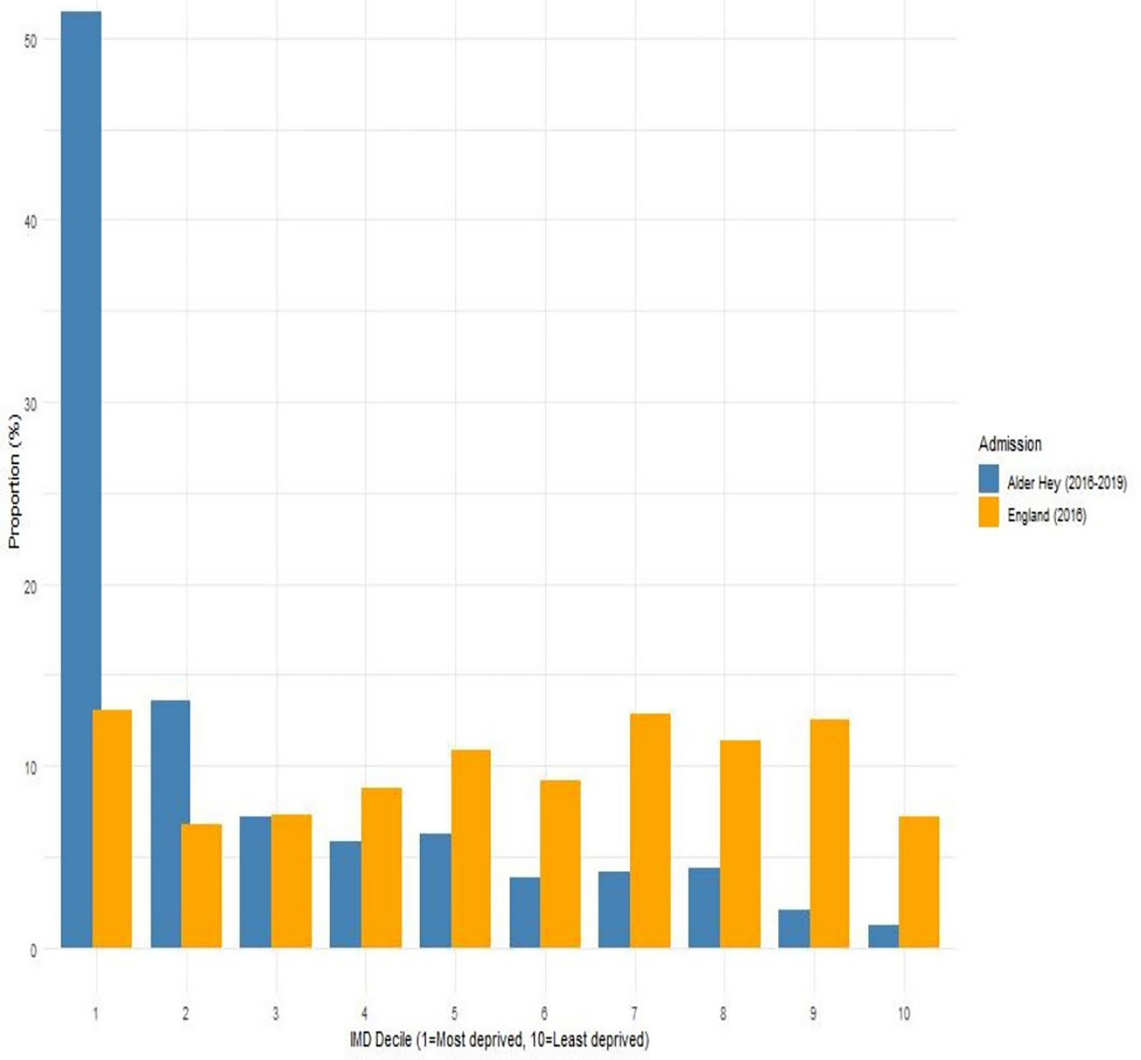
Emergency admissions (0–18 years) for Liverpool and England by IMD decile. IMD, Index of Multiple Deprivation.

**Figure 2:**
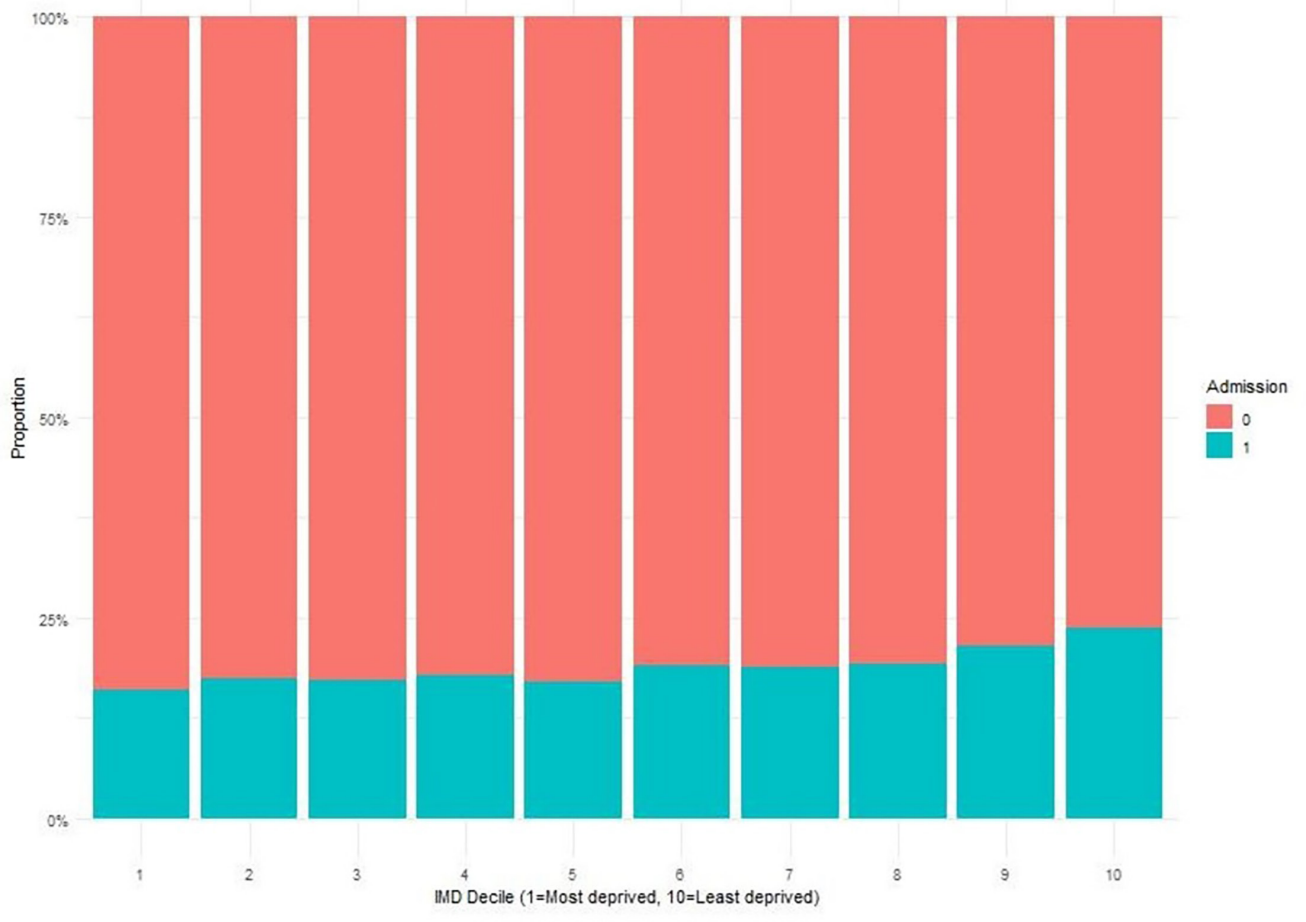
Proportion of attending patients who were admitted from the ED by IMD. ED, emergency department; IMD, Index of Multiple Deprivation.

Online supplemental figure S1 presents a flow chart of the included participants for analysis. After applying exclusion criteria and restricting to observations with complete data, the final analytical sample comprised 206 918 attendances between 1 January 2016 and 31 December 2019, with 93 219 unique paediatric patients. Of these attendances, there were 34 952 (17%) admissions from 24 043 unique patients. Some children attended the ED multiple times during the study period, with a maximum of 44 attendances per child. Figure 1 illustrates the distribution of socio-economic disadvantage from all admissions within the study population compared with the rest of England in 2016/2017. 51% of total admissions came from the most disadvantaged decile, compared with 13% from England (in 2016). Figure 2 illustrates the proportion of attendances that resulted in admission by socio-economic disadvantage group. Approximately 16% of all attendances from the most disadvantaged group resulted in admission, compared with 24% in the least disadvantaged group. Therefore, despite children from the most disadvantaged groups accounting for more attendances and admissions overall, the proportion of attenders being admitted from the ED increased in a graded fashion from the most disadvantaged to the least disadvantaged group. Online supplemental figures S3–S5 show further distributions of age, time spent in the ED and number of visits per patient.

The median ED attendance duration was 2.4 hours (IQR 1.5–3.3) and 3.1 hours (IQR 1.9–3.8) for all attendances and admissions, respectively. Inpatient duration of less than 1 day accounted for 55% of all admissions (34 592 admissions). The median age for the total attendees was 3.6 years (IQR 1.3–9.1 years). Approximately 46% of the patients were female. The number of further emergency attendances and admissions was counted for each patient. Due to data limitations, we were unable to distinguish whether those events happened (1) after the initial ED visit but before any hospital admission, or (2) after the patient was discharged from their inpatient stay (if they were admitted).

There was a total of 44 511 reattending patients, and a maximum of 44 attendances. Children in the most disadvantaged decile accounted for 55% of attendances and 51% of admissions compared with 1% of attendances and 1% of admissions in the least disadvantaged decile.

Online supplemental table S2 presents prevalence of risk factors and main hospital outcomes (attendance, admission and length of stay) of the study population (all ED attendances), stratified by IMD decile. The more disadvantaged children were likely to have fewer investigations performed, be triaged as ‘Green’ or ‘GP appropriate’, attend the ED out of hours, be self-referrals, have shorter inpatient stay and have diagnoses of infectious disease and respiratory conditions compared with their most affluent counterparts.

### Main model results

The GAM shows a monotonic relationship between IMD score (as a continuous measure) and the risk of admission, where children from the more disadvantaged areas have a smaller likelihood of admission than those from the least disadvantaged (online supplemental figure S6). Further, the nature of this relationship provides justification to treat IMD as a continuous variable within logistic regression, rather than a grouped variable. Online supplemental figure S7 displays the logit of the predicted probabilities against the continuous IMD score.

To examine the relationship between SECs and likelihood of admission, conditional on attendance, three logistic regression models were run (see table 1). Model 1 presents the unadjusted association between odds of admission and low SECs, Model 2 additionally adjusts for demographic variables (age, gender, season and arrival year) and Model 3 presents additionally adjusted for case-mix and clinical presentation variables that may lie on the pathway between socio-economic circumstances and admission (as described in the Directed Acylic Graph (DAG)).

**Table 1:** Results of logistic regression showing the association between odds of admission and IMD

Model	IMD OR	Lower 95% CI	Upper 95% CI	P value
Model 1: Unadjusted	0.996	0.995	0.996	<0.05
Model 2: Confounder adjusted	0.996	0.995	0.996	<0.05
Model 3: Adjusted for confounders, case-mix and clinical presentation variables	1.002	1.000	1.003	<0.05

Data based on 206 918 observations.

IMD, Index of Multiple Deprivation.

Model 1 showed that increasing deprivation was associated with lower odds of admission conditional on ED attendance (OR per one-unit increase in IMD 0.996, 95% CI 0.995 to 0.996). Although the per-unit effect size appears small, IMD scores ranged broadly across the cohort (approximately 0–80). Consequently, this corresponded to substantially lower odds of admission among children living in the most deprived areas compared with those living in the least deprived areas. For example, as an approximation, these results suggest that children from the most deprived areas were 27% less likely to be admitted compared with their most affluent counterparts. After adjusting for demographic variables (age, gender, season and arrival year), this association did not change (Model 2). After additionally adjusting for case-mix and clinical presentation variables, the direction of association reversed, with increasing deprivation associated with slightly higher odds of admission (Model 3). Note, all models can be found in online supplemental table S3-S5.

### Results describing the relationship between risk factors of admission and SECs

Online supplemental table S6 presents univariate associations between SECs and other important variables in this analysis, across all attendances and all admissions respectively. Note, analyses of inpatient length of stay were descriptive and were not adjusted for potential confounders. Therefore, these findings should be interpreted cautiously and not as evidence of causal relationships.

Lower SECs was significantly associated with inpatient stay, diagnosis, referral category, triage priority, investigation count and time spent in the ED for all attendances and admissions. Out of hours attendance and year were only associated with lower SECs within attendances to the ED.

**Figure 3:**
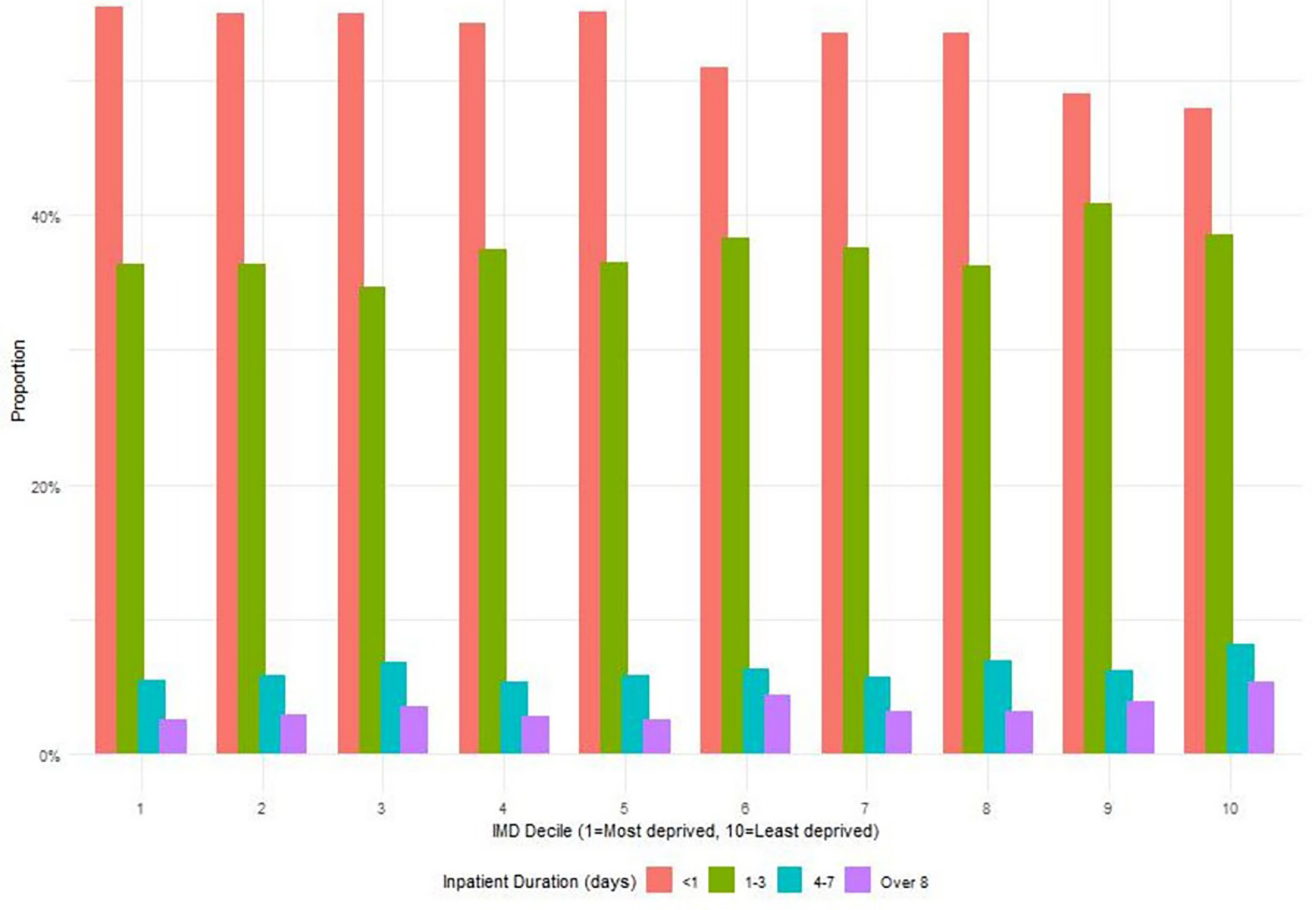
Proportion of inpatient length of stay for all admissions, by IMD. IMD, Index of Multiple Deprivation.

**Figure 4:**
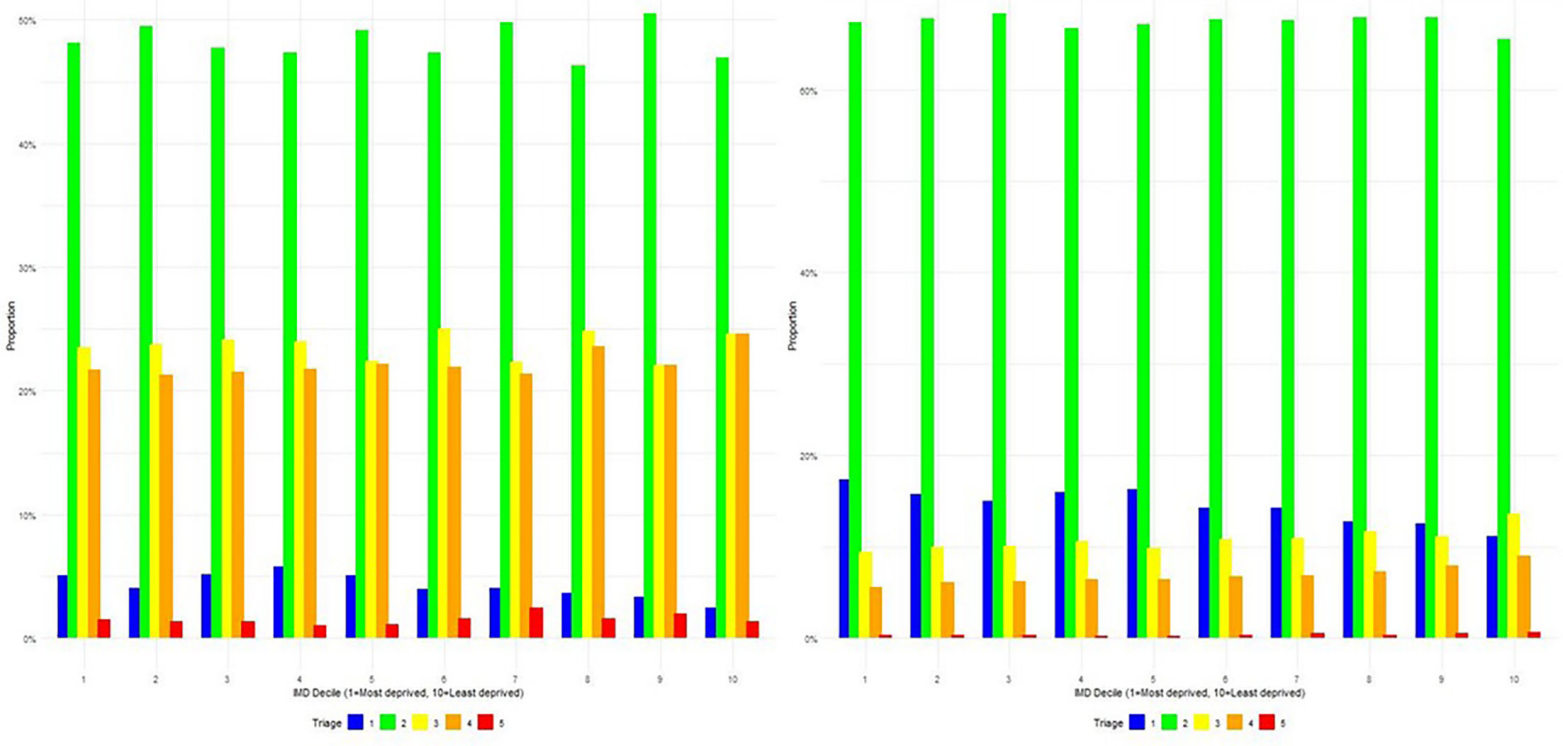
Proportion of triage category for all admissions and attendances, by IMD. IMD, Index of Multiple Deprivation.

The least deprived children had longer in patient stays and the most disadvantaged children had higher proportions of shorter inpatient stays (less than 1 day) (figure 3). To explore if this relationship can be explained by markers of disease acuity, the distribution of triage categories across IMD groups, for all attendances and all admissions, respectively were examined (see figure 4). Of all attendances, the most deprived children were more likely to be triaged as ‘GP appropriate’ and ‘Green’ than their most affluent peers. The most advantaged children were more likely to be triaged as ‘Yellow’ and ‘Orange’ compared with their least affluent peers, suggesting these children had higher acuity attendances. Both groups had similar proportions of attendances triaged as ‘Red’. A similar pattern can be seen for all admissions.

Admissions due to infectious disease and respiratory conditions were more common among the most disadvantaged children, with a graded decrease in proportion of respiratory conditions as IMD advantage increased. The most advantaged children had a higher proportion of attendances for gastrointestinal conditions than their least advantaged peers. Overall, self-referrals accounted for most ED attendances across all IMD groups, with a decreasing gradient as SECs increased. The opposite trend was observed for hospital referrals and for referrals from within Alder Hey, where more affluent children were more likely to be referred to the ED via these routes. Child attendees living in areas of lower SECs were more likely to have been referred by out of hours care (including other primary care which does not require an appointment) than their most affluent peers. However, proportions of these referrals for the most disadvantaged children were similar in children from the middle IMD groups. Proportions of referrals from primary care services remained generally consistent across IMD deciles. The proportion of all other variables by IMD decile for all attendances and admissions, respectively, can be found in online supplemental figures S8–S15.

The majority of attendances did not have an investigation performed. For all attendances, the least deprived children were more likely to have one or more investigations performed compared with the most deprived, and the most deprived were most likely to have no investigations performed. A similar trend can be observed for all admissions.

Overall, there were more attendances during out of hours than during normal working hours. The most deprived children were more likely to attend the ED out of hours than their most affluent peers, and the most affluent children were more likely to attend during normal hours compared with the least affluent children.

### Sensitivity analyses

Results using GEEs were broadly consistent with the direction of estimates observed in unadjusted analyses (see table 2). However, after additionally adjusting for case-mix and clinical presentation variables, the association between low SECs and admission attenuated towards the null in the GEE models (OR=1), suggesting the observed association may be sensitive to the method used to account for repeated attendances. The retained rounded output gave an OR of 1.000 with confidence limits also rounding to 1.000 at three decimal places, so this estimate should be interpreted as evidence of attenuation rather than as an exact null estimate. Differences between approaches may reflect the influence of repeated attendances among high-frequency ED users, which can affect estimates when observations are not fully independent.

**Table 2:** Results of GEEs showing the association between odds of admission and IMD

Model	IMD OR	Lower 95% CI	Upper 95% CI	P value	Sig
Model 1 (unadjusted)	0.996	0.995	0.996	<0.05	^***^
Model 2 (confounder adjusted)	0.995	0.995	0.996	<0.05	^***^
Model 3 (confounder and mediator adjusted)	∼1.000	∼1.000	∼1.000	–	–

Data based on 206 918 observations.

Values are reported from retained rounded model outputs. For the fully adjusted GEE model, the estimate and CI rounded to 1.000 at three decimal places; therefore, this result is interpreted as attenuation towards the null rather than evidence of a precise null effect.

***p<0.01, **p<0.05, *p<0.1

GEE, generalised estimating equation; IMD, Index of Multiple Deprivation.

## Discussion

We investigated inequalities in the risk of hospital admission within a tertiary ED in an area of high SEC disadvantage in England and provide insight into the social patterning of other important factors, such as referral source, diagnosis, triage category and investigations performed. We made use of a rich dataset of individual-level patient data during their ED attendance, including demographic and clinical information. We show that more advantaged children are more likely to be admitted to hospital from ED, conditional on attendance to ED. However, these children presented with higher triage acuity and differing clinical characteristics, and after adjustment of case-mix and clinical presentation variables, the picture changes. After adjusting for case-mix clinical presentation variables, it is the most deprived children that are more likely to be admitted from the ED, compared with their more affluent peers, despite these children presenting with lower triage acuity and fewer investigations performed.

We support previous UK findings that ED attendance was more common among the most disadvantaged groups,^42^ and that SEC disadvantage is associated with admissions.^1642^,^45^ However, we provide unique insight into the association between SEC disadvantage and paediatric ED admission, conditional on attendance. The crude association between SEC disadvantage and risk of admissions showed approximately 27% lower odds among the most deprived to be admitted than their least affluent peers. Even after adjusting for confounding factors (age, gender, arrival year and season), this association remained consistent. On adjustment of case-mix and clinical presentation variables that may lie on the pathway between socio-economic circumstances and admission, this reversed, resulting in children from the most disadvantaged areas experiencing a 20% increased likelihood of admission, compared with their most affluent counterparts. This substantial attenuation and reversal after adjustment may reflect differences in clinical presentation and care pathways across socio-economic groups.

The most disadvantaged children attending the ED presented as less acutely unwell in terms of triage and other measures. They were more likely to have fewer investigations performed, be triaged as ‘Green’ (‘GP appropriate’), attend the ED out of hours, be self-referrals and referrals from other primary care (not requiring an appointment) compared with their most affluent counterparts. We also reflect findings that the most deprived children accounted for more admissions due to common conditions have higher short stay admissions due to respiratory infections.^4 5^

Higher proportions of shorter inpatient stays (less than 1 day) among the most disadvantaged children could reflect findings that a significant proportion of admissions from the ED could be affected by issues other than medical acuity such as inadequate access to primary care^46^ and differences in parental management of children’s illness at home in the most deprived groups.^47^

Despite evidence of higher triage acuity as a risk factor for admission,^22 24 48^ our findings show that the most disadvantaged children are more likely to be considered lower triage acuity compared with their most affluent counterparts (described elsewhere as non-urgent paediatric ED attendances^20^) may reflect differences in clinical decision-making, risk tolerance or unmeasured social factors influencing admission, potentially reflecting precautionary admission practices for socially vulnerable children.

We support findings of referral source as an influence on risk of admissions.^22 24 27^ Higher proportions of referrals by another hospital or within Alder Hey itself among the most affluent children may reflect increased markers of disease acuity, thereby hospitals referring patients to a tertiary hospital with specialist expertise. It may also reflect increased parental advocacy for urgent care within the most affluent groups, and it is plausible that ED clinicians may be more likely to expect more severe presentations from those referred from other hospitals, while also anticipating self-referrals to most likely be ‘Green’ attendances. Further, higher proportions of self-referrals from the most disadvantaged children may reflect inequalities in scheduled elective care, where people living in the most deprived areas are twice as likely to wait more than a year for non-urgent treatment.^49^ The Darzi Report published in 2024, commissioned by the UK government to identify the key drivers behind the National Health Service’s current challenges and to inform the development of a 10-year strategy for reforming the health service, also indicates that children account for approximately 80% of people waiting over a year for community health services^50^ and disadvantaged communities may be disproportionately affected by this.

To our knowledge, this is the first analysis to examine the relationship between SEC disadvantage and risk of emergency hospital admission in children presenting to the ED, using individual level data within a tertiary paediatric setting. We used logistic regression models to estimate associations between SECs and admission risk. We made use of a rich dataset, including demographic and clinical information on approximately 93 000 patients across 4 years.

This study had several potential limitations that should be considered as they may have influenced the results and should be addressed in future research. We presented estimates between SECs and hospital admission conditional on attending the ED which are not reflective of the general relationship between SECS and emergency hospital admissions across England. The catchment area for the ED is primarily Liverpool city which experiences lower SECs than the English average, which may introduce selection bias,^51 52^ and therefore the regression equations in this study are derived from a specific set of patients. While findings may be generalisable to similar child populations in urban areas of low SECs, the characteristics may differ to child populations in different settings. Our hospital is the main provider of acute paediatric services in Liverpool and therefore the data should have captured most paediatric admissions, but nonetheless a single site study still introduces limitations. Methods used in this study should be replicated in other geographical areas with differing levels of social disadvantage, and across paediatric and mixed EDs to examine the generalisability of findings. Residual confounding and potential selection bias may remain, and causal interpretations cannot be inferred from this observational analysis. We made the best use of the available data to capture plausible mechanisms through which low SECs may influence hospital outcomes. However, there is a possibility of the existence of unmeasured or uncontrolled confounding variables that may have impacted these results. This could lead to an incomplete understanding of the true relationship between the variables of interest. Information on vaccination status, prevalence of chronic/complex illness and prematurity are some examples of this. Finally, the association between socio-economic disadvantage and admission was sensitive to modelling strategy. Although logistic regression models with cluster-robust SEs and GEE models produced broadly similar directional findings, the fully adjusted GEE estimates attenuated towards the null. This may reflect differences between subject-specific and population-averaged estimation approaches, as well as the influence of repeated attendances among high-frequency ED users. Consequently, the magnitude of association should be interpreted cautiously.

While our findings are likely to remain highly relevant to current (postpandemic) ED attendance, repeated methods on post-pandemic data could further assess the most recent relationship between SECs and the care pathways to paediatric ED admission, to further explore if the associations found in this study have been impacted after the pandemic, where communities who were already disadvantaged were further disproportionately affected by lockdown measures. Other factors which may be associated with emergency admission, such as ethnicity^24^ and the existence of ethnic inequalities in paediatric healthcare utilisation,^53^ vaccination status and gestation period,^21 54^ were not studied here. Further studies should examine other potential predictors of ED admission and their relationship with low SECs. Finally, findings suggest that there may be more complex pathways to ED admission at play, that may have not been captured here. Qualitative studies with parents and clinicians focusing on the effects of socio-economic disadvantage could provide further insight and solutions for mitigating the effects found here and could also provide context into the evidence found in this study suggestive of potential differences in clinical decision-making relating to social vulnerability within an ED setting.

In conclusion, this study investigated inequalities in the risk of paediatric ED admission within a tertiary ED in an area of low SECs in England. It further provides insight into the social patterning of other important factors, such as referral source, diagnosis, triage category and investigations performed. We identify a complex relationship between low SECs and risk of admission in children, showing the influence of a child’s clinical condition and referral pathways on increased likelihood of admission. This study suggests that low SECs are associated with risk of paediatric ED admissions; however, the relationship is complex and influenced by several factors such as referral pathways and triage. This study supports existing evidence of clinical and triage information as risk factors for ED admission and provides further insight into the social patterning of paediatric ED attendances in a tertiary setting. Patients from the most disadvantaged deciles accounted for more attendances, including those from out of hours care and self-referrals. This suggests that the increasing trends in hospital admissions are not necessarily inexorable and interventions aiming to reduce unnecessary emergency hospital use, especially for children with low disease acuity, should focus on communities experiencing low SECs, to ensure equitable access to services and safety netting advice. The findings contribute to our understanding of the factors influencing paediatric admissions, and the associations between patient characteristics and diagnostic information and further provide a more detailed picture of the patient pathway.

## Supplementary material

10.1136/bmjph-2025-002916 online supplemental file 1

## Data Availability

No data are available.
