# Supplementary material for: Assessing inequalities in paediatric emergency department admissions in the Northwest of England: a retrospective cohort study
Source: BMJ Public Health. 2026 Jun 19;4(2):e002916. doi: 10.1136/bmjph-2025-002916 (PMC13289404; doi:10.1136/bmjph-2025-002916)

Supplementary File

Figure S1: Flow chart of included patients

The original dataset included 331,591 attendances over a 5-year period between mid-2015 to mid-2020. Since we analysed IMD score, patients without an available postcode were omitted (approximately 2,400 observations, 1,800 patients). Due to potential disruptions caused by the COVID-19 pandemic, data from 2020 were excluded. Additionally, data from 2015 were removed for consistency and simplicity in the dataset. Any patient listed was included regardless of any diagnosis, provided they had available postcode data to link to IMD scores. This resulted in a total of 240,653 attendances. Complete data for all variables analysed in this study resulted in a final dataset of 206,918 attendances (86%).

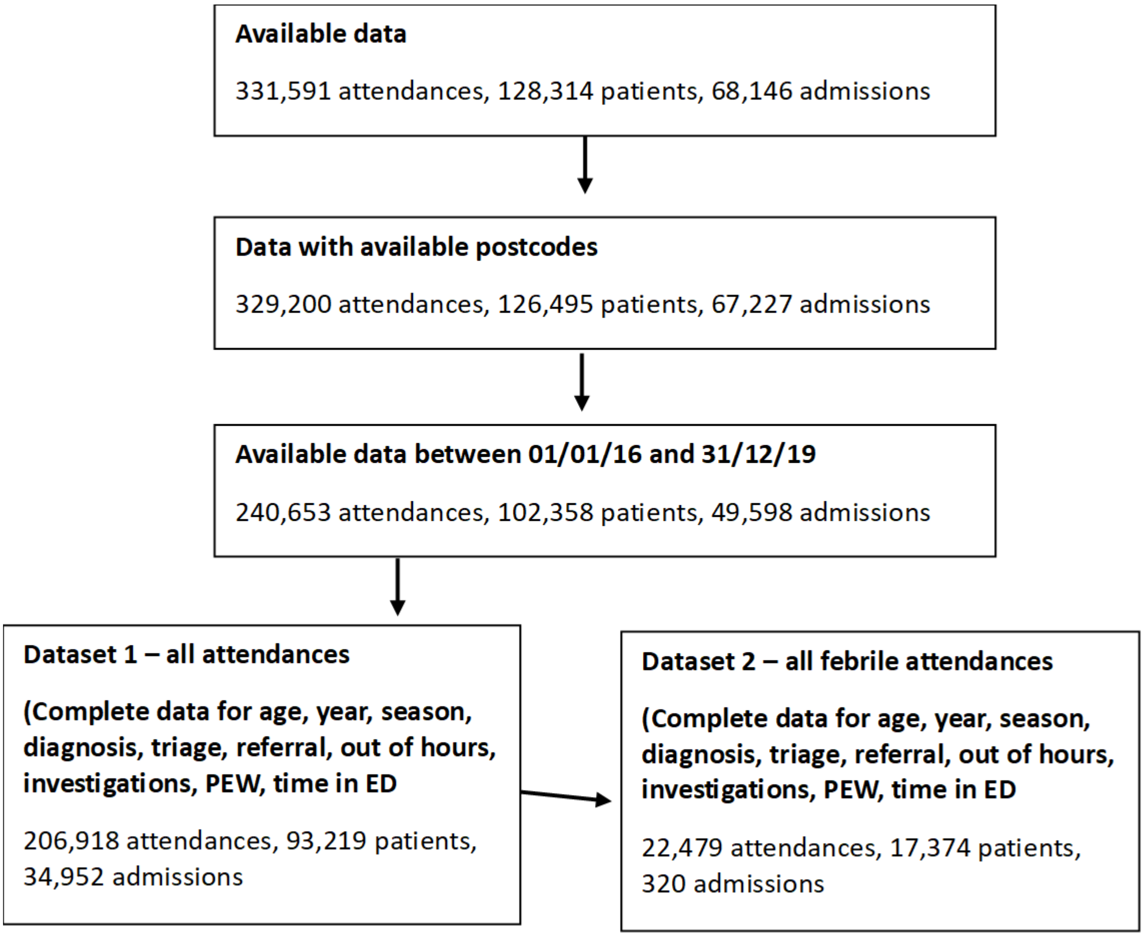

Figure S2: Logic model

This figure illustrates our hypothesised causal pathway from small area deprivation to paediatric hospital admission, conditional on attendance. The model assumes that deprivation may influence the likelihood of hospital admission both directly and indirectly through its effect on disease severity measures. Disease severity measures include proxy measures of disease severity and case-mix factors, namely diagnosis, MTS triage system, type of referral, out of hours arrival, number of investigations and time spent (hours) in the ED. We also adjusted for baseline variables—age, sex, year, and season—as they may influence the outcome and our disease severity measures as encoded in the logic model. Our logic model informed our sequential regression modelling to assess whether the association between deprivation and admission is attenuated after accounting for disease severity or case-mix mediators.

Note: Dashed line represents our exposure variable (small area deprivation) and dotted lines represent baseline confounder variables.

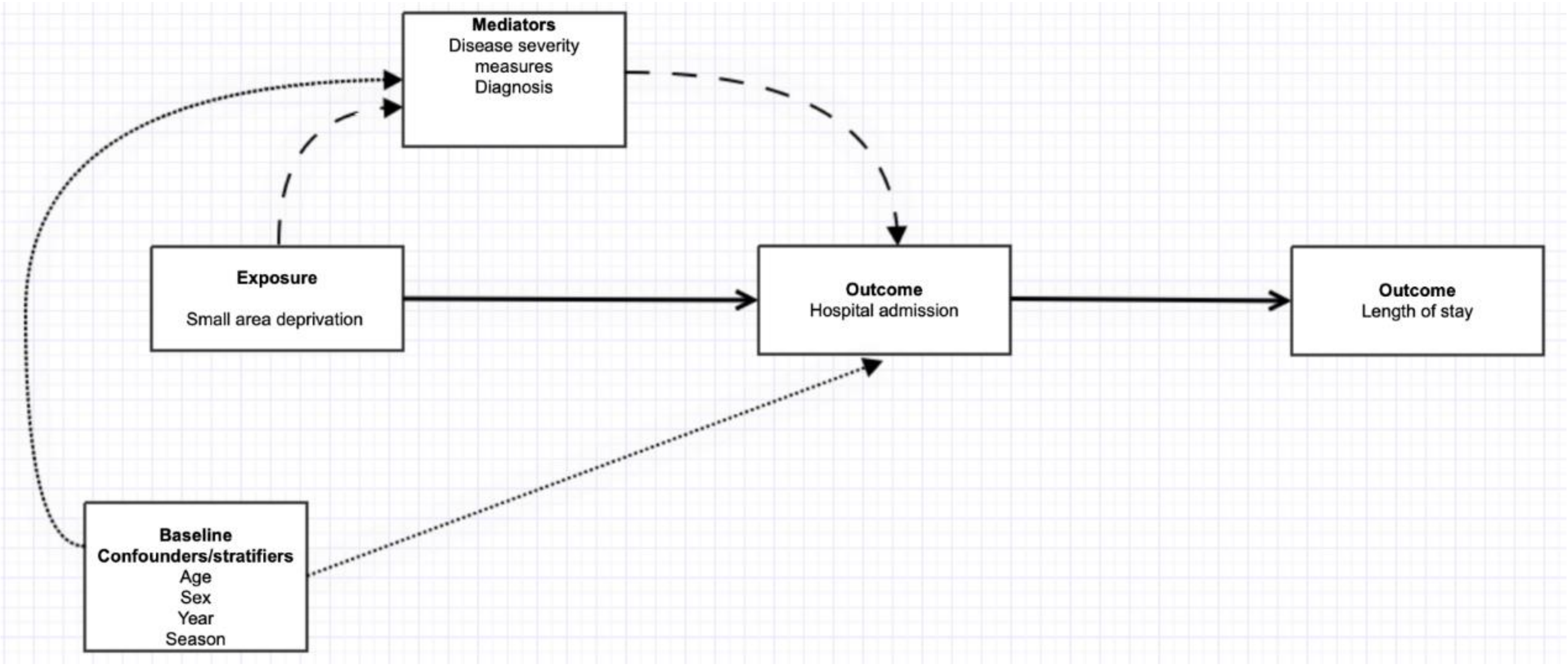

Table S1: Data sources and measures

Variable name	Description
Admission	A binary outcome variable indicating if the patient attending the ED was subsequently admitted or not.
Length of stay	The duration, in hours, between time of arrival and discharge, irrespective of admission.
Inpatient stay	Inpatient length of stay was measured as the duration between admission and discharge (days).Categories are less than one day (1); between 1 and 3 days (2); between 4 and 7 days (3); 8 days and over (4).
Study number	The patient's individual identifier
Attendance Number	The patient's attendance identifier.
Age	The age of the patient at arrival (years). A continuous age variable, indicating age in years, was derived using date of birth, and arrival date information.
Age group	Group variable of the age of the patient upon arrival:<br><3 months, 3 to <12 months, 1 to <6 years, 6 to <12 years, 12+ years
Gender	Factor variable, indicating the patient's gender (male or female).
IMD decile	Index of Multiple Deprivation decile
IMD score	Index of Multiple Deprivation score
Triage priority	The category each patient had at triage which represents the initial evaluation of a child using the Manchester Triage System (MTS). Patients who triaged MTS 'Green', without any complicating factors or comorbidities, and presenting with conditions that were deemed suitable to be treated by a GP were labelled 'GP appropriate'. (1-5).<br>1 = GP, 2 = GREEN, 3 = YELLOW, 4 = ORANGE, 5 = RED
Out of hours	A logical variable indicating if the patient arrived at hospital during out of hours. Out of hours attendance was defined by any attendance occurring between 6pm and 8am, and anytime at weekends to account for any increased attendances due to inability to access other healthcare services.
Season	To account for these seasonal trends, where hospitals may often experience increased attendances due to respiratory infections (like influenza and pneumonia) in colder months, we derived (from arrival month) a variable indicating the season the patient attended hospital. Variable indicates the season in the year the patient arrived. Spring (March – May), Summer (June – August), Autumn (September – November), Winter (December - February).

Arrival year	The year the patient arrived in the ED
Referral	A categorical variable, indicating how the patient was referred to the ED. Each referral organization assigned to each patient upon their attendance was further condensed into five categories: Hospital (1); Primary care (including all referrals from GP practices, dentists and other forms of primary care) (2); Out of hours (including all referrals from healthcare services that can be contacted out of hours or without an appointment) (3); 'Self', where no previous contact with health services was assumed (4); 'Alder Hey' (5). The latter was used as a referral source within the dataset. Upon discussion with clinicians, this could include a broad range of sources and was therefore coded as a separate category. This may have included, self-referrals to Alder Hey, attendance via ambulance and may also indicate any patients within Alder Hey who were referred to the ED.
Investigation count	Variable describes how many investigations (e.g. blood tests, urine samples and X-ray) were taken for each patient (0-3). Information on the investigations performed (e.g. X-rays, blood tests, and urine tests) for each patient were open to free text from clinicians. On consultation with clinicians , we decided that information regarding how many investigations were conducted, was more useful than the details of each investigation. The maximum observed number investigations within an attendance were 3. Therefore, I derived a new variable, indicating the number of investigations performed for each patient, 0 to 3.
Diagnosis	Variable indicating the primary diagnosis of the patient. Original information on diagnoses and sub diagnoses was vast, and open to free text for clinicians. The data did not use ICD-10 codes. For simplicity, these were coded into seven categories: Central nervous system (1); ENT (Ear nose throat) conditions (2); Gastrointestinal conditions (3); Infectious disease (4); Respiratory conditions (5); Urological conditions (6); and other conditions (7). These were grouped in this way to maintain similar original diagnoses labelling that was indicated within the clinician's free text.
PEW score (PEWS)	The PEWS range, related to sepsis concern, indicated in clinician notes. This variable included four categories: PEWS under 2 (1); PEWS 2 to 7 (2); PEWS 7 to 13 (3); and PEWS 13 and over.

Figure S3: Histogram of attendances per patient (2016-2019)

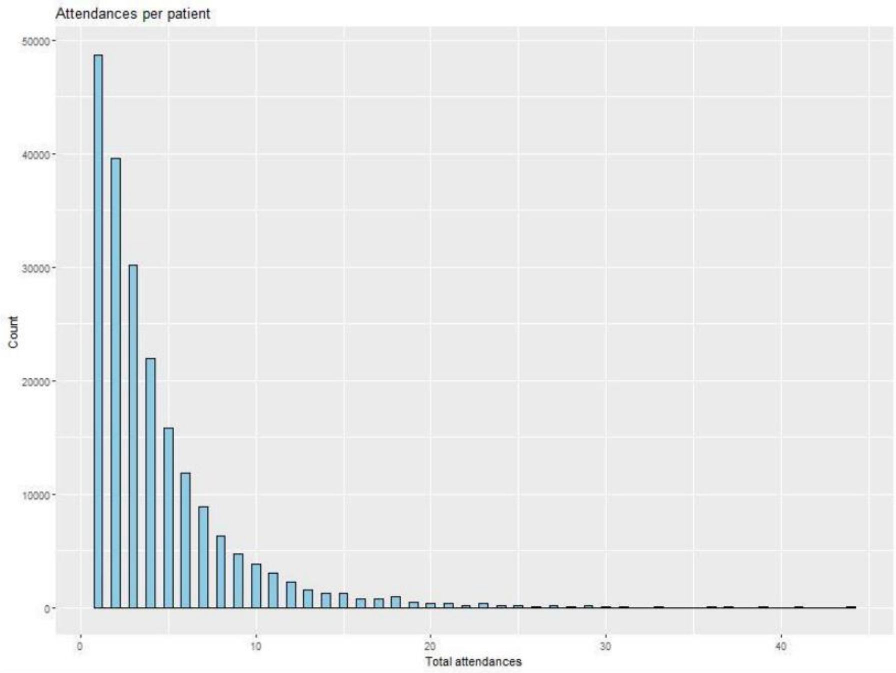

Figure S4: Histogram of time (hours) spent in the Emergency Department per visit

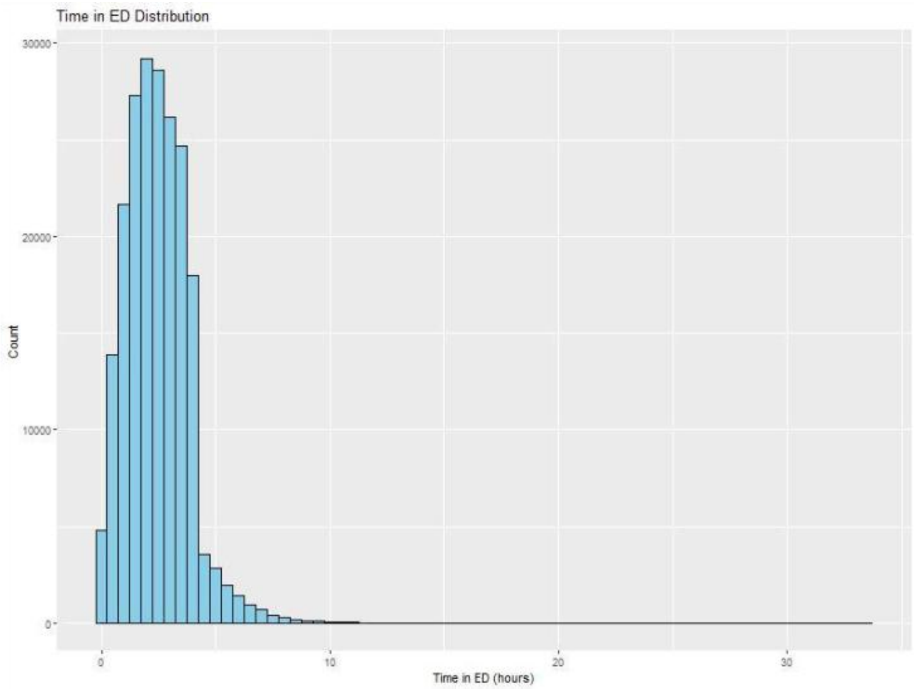

Figure S5: Histogram of age (years) of attending patients

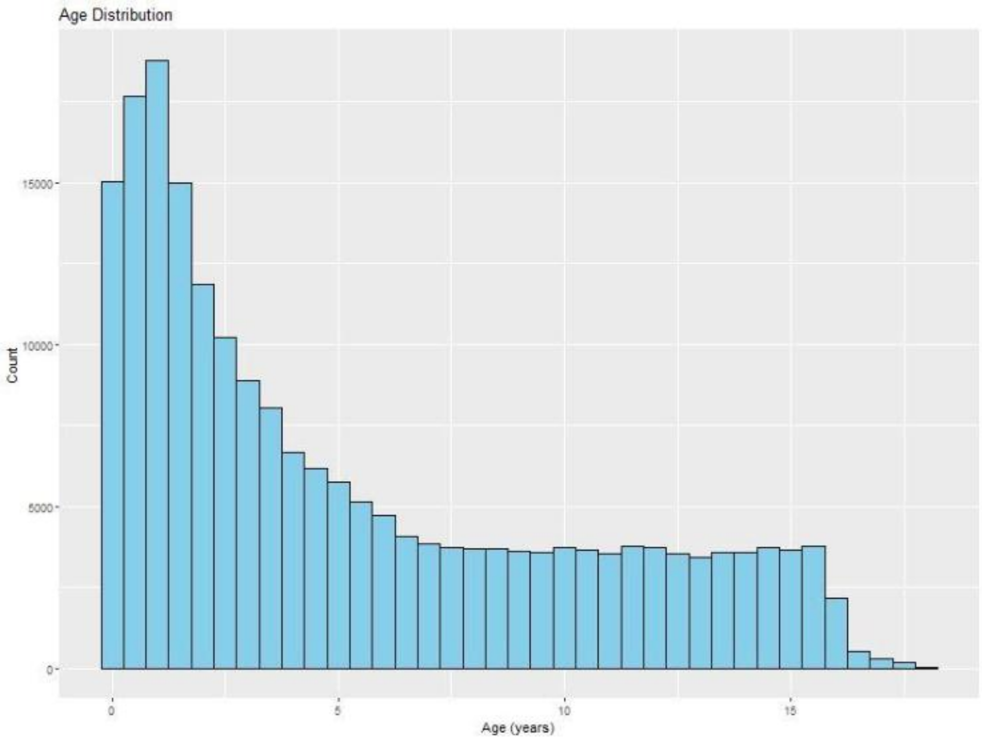

Table S2: Prevalence of risk factors and main hospital outcomes

IMD Decile	1 (Most deprived)	2	3	4	5	6	7	8	9	10 (Least deprived)	p for trend
Attendances	112771	27347	14479	11509	12892	7030	7648	7952	3421	1869	
Admissions	17975 (15.9%)	4742 (17.3%)	2498 (17.3%)	2045 (17.8%)	2202 (17.1%)	1345 (19.1%)	1437 (18.8%)	1526 (19.2%)	739 (21.6%)	443 (23.7%)	<0.001
Triage											<0.001
GP	19623 (17.4%)	4300 (15.7%)	2176 (15.0%)	1836 (16.0%)	2107 (16.3%)	1005 (14.3%)	1087 (14.2%)	1013 (12.7%)	428 (12.5%)	208 (11.1%)	
Green	75882 (67.3%)	18544 (67.8%)	9883 (68.3%)	7673 (66.7%)	8654 (67.1%)	4755 (67.6%)	5164 (67.5%)	5397 (67.9%)	2322 (67.9%)	1224 (65.5%)	
Yellow	10635 (9.4%)	2743 (10.0%)	1465 (10.1%)	1227 (10.7%)	1270 (9.9%)	764 (10.9%)	834 (10.9%)	932 (11.7%)	382 (11.2%)	255 (13.6%)	
Orange	6288 (5.6%)	1677 (6.1%)	910 (6.3%)	745 (6.5%)	829 (6.4%)	478 (6.8%)	523 (6.8%)	583 (7.3%)	271 (7.9%)	169 (9.0%)	
Red	343 (0.3%)	83 (0.3%)	45 (0.3%)	28 (0.2%)	32 (0.2%)	28 (0.4%)	40 (0.5%)	27 (0.3%)	18 (0.5%)	13 (0.7%)	
Arrival age	5.29 (4.78)	5.37 (4.85)	5.58 (4.90)	5.30 (4.86)	5.29 (4.85)	5.75 (5.02)	5.56 (4.88)	5.61 (4.92)	5.88 (5.03)	5.84 (4.96)	<0.001
Male	60693	14842	7951	6309	6928	3738	4463	4353	1941	1057 (56.6%)	<0.001

	(53.8%)	(54.3%)	(54.9%)	(54.8%)	(53.7%)	(53.2%)	(58.4%)	(54.7%)	(56.7%)		
Number of investigations											<0.001
0	85418 (75.7%)	20423 (74.7%)	10666 (73.7%)	8484 (73.7%)	9657 (74.9%)	5125 (72.9%)	5574 (72.9%)	5724 (72.0%)	2412 (70.5%)	1298 (69.4%)	
1	16648 (14.8%)	4063 (14.9%)	2262 (15.6%)	1771 (15.4%)	1888 (14.6%)	1110 (15.8%)	1093 (14.3%)	1255 (15.8%)	559 (16.3%)	297 (15.9%)	
2	3600 (3.2%)	999 (3.7%)	527 (3.6%)	428 (3.7%)	429 (3.3%)	261 (3.7%)	285 (3.7%)	274 (3.4%)	134 (3.9%)	88 (4.7%)	
3	7105 (6.3%)	1862 (6.8%)	1024 (7.1%)	826 (7.2%)	918 (7.1%)	534 (7.6%)	696 (9.1%)	699 (8.8%)	316 (9.2%)	186 (10.0%)	
Length of stay	2.46 (1.35)	2.46 (1.35)	2.44 (1.34)	2.43 (1.34)	2.43 (1.33)	2.45 (1.35)	2.47 (1.36)	2.46 (1.35)	2.49 (1.39)	2.42 (1.37)	<0.001
Season											0.302
Spring	29281 (26.0%)	7066 (25.8%)	3671 (25.4%)	2974 (25.8%)	3330 (25.8%)	1750 (24.9%)	2009 (26.3%)	2046 (25.7%)	911 (26.6%)	488 (26.1%)	
Summer	24807 (22.0%)	6119 (22.4%)	3196 (22.1%)	2540 (22.1%)	2854 (22.1%)	1621 (23.1%)	1687 (22.1%)	1714 (21.6%)	796 (23.3%)	449 (24.0%)	
Autumn	29915 (26.5%)	7192 (26.3%)	3846 (26.6%)	3099 (26.9%)	3423 (26.6%)	1845 (26.2%)	1999 (26.1%)	2201 (27.7%)	858 (25.1%)	466 (24.9%)	
Winter	28768 (25.5%)	6970 (25.5%)	3766 (26.0%)	2896 (25.2%)	3285 (25.5%)	1814 (25.8%)	1953 (25.5%)	1991 (25.0%)	856 (25.0%)	466 (24.9%)	

OOH arrival	65217 (57.8%)	15780 (57.7%)	8236 (56.9%)	6526 (56.7%)	7455 (57.8%)	4009 (57.0%)	4398 (57.5%)	4638 (58.3%)	1900 (55.5%)	1033 (55.3%)	0.007
Referral group											<0.001
Primary care	6990 (6.2%)	1688 (6.2%)	897 (6.2%)	797 (6.9%)	833 (6.5%)	543 (7.7%)	579 (7.6%)	513 (6.5%)	231 (6.8%)	108 (5.8%)	
Hospital	1039 (0.9%)	304 (1.1%)	167 (1.2%)	152 (1.3%)	119 (0.9%)	117 (1.7%)	123 (1.6%)	117 (1.5%)	65 (1.9%)	45 (2.4%)	
Alder Hey	18492 (16.4%)	4926 (18.0%)	2567 (17.7%)	2129 (18.5%)	2288 (17.7%)	1393 (19.8%)	1540 (20.1%)	1683 (19.9%)	789 (23.1%)	466 (24.9%)	
OOH	11581 (10.3%)	3025 (11.1%)	1444 (10.0%)	1129 (9.8%)	1494 (11.6%)	719 (10.2%)	768 (10.0%)	831 (10.5%)	274 (8.0%)	124 (6.6%)	
Self	74669 (66.2%)	17404 (63.6%)	9404 (64.9%)	7302 (63.4%)	8158 (63.3%)	4259 (60.6%)	4638 (60.6%)	4908 (61.7%)	2062 (60.3%)	1126 (60.2%)	
Diagnosis											<0.001
Infectious disease	15635 (13.9%)	3598 (13.2%)	1862 (12.9%)	1550 (13.5%)	1746 (13.5%)	892 (12.7%)	1007 (13.2%)	982 (12.3%)	404 (11.8%)	206 (11.0%)	
Respiratory conditions	23035 (20.4%)	5404 (19.8%)	2743 (18.9%)	2198 (19.1%)	2468 (19.1%)	1314 (18.7%)	1481 (19.4%)	1521 (19.1%)	557 (16.3%)	253 (13.5%)	
Urological conditions	2725 (2.4%)	728 (2.7%)	379 (2.6%)	339 (2.9%)	340 (2.6%)	196 (2.8%)	231 (3.0%)	231 (2.9%)	116 (3.4%)	68 (3.6%)	
GI conditions	11527 (10.2%)	2864	1399	1268	1288	732	804	764	360	229 (12.3%)	

		(10.5%)	(9.7%)	(11.0%)	(10.0%)	(10.4%)	(10.5%)	(9.6%)	(10.5%)		
ENT conditions	3617 (3.2%)	853 (3.1%)	443 (3.1%)	328 (2.8%)	407 (3.2%)	211 (3.0%)	235 (3.1%)	229 (2.9%)	126 (3.7%)	62 (3.3%)	
CNS	1472 (1.3%)	346 (1.3%)	226 (1.6%)	158 (1.4%)	171 (1.3%)	78 (1.1%)	115 (1.5%)	132 (1.7%)	54 (1.6%)	33 (1.8%)	
Other	54760 (48.6%)	13554 (49.6%)	7427 (51.3%)	5668 (49.2%)	6472 (50.2%)	3607 (51.3%)	3775 (49.4%)	4093 (51.5%)	1804 (52.7%)	1018 (54.5%)	
PEW score (PEWS)											0.238
Under 2	112725 (100.0%)	27332 (99.9%)	14476 (100%)	11501 (99.9%)	12886 (100%)	7026 (99.9%)	7644 (99.9%)	7945 (99.9%)	3419 (99.9%)	1869 (100%)	
2 to 7	31 (0.0%)	8 (0.0%)	2 (0.0%)	4 (0.0%)	4 (0.0%)	4 (0.1%)	4 (0.1%)	7 (0.1%)	1 (0.0%)	0 (0.0%)	
7 to 13	14 (0.0%)	7 (0.0%)	1 (0.0%)	4 (0.0%)	1 (0.0%)	0 (0.0%)	0 (0.0%)	0 (0.0%)	1 (0.0%)	0 (0.0%)	
13 and over	1 (0.0%)	0 (0.0%)	0 (0.0%)	0 (0.0%)	1 (0.0%)	0 (0.0%)	0 (0.0%)	0 (0.0%)	0 (0.0%)	0 (0.0%)	
Inpatient stay											<0.001
Less than 1 day	9960 (55.4%)	2604 (54.9%)	1373 (55.0%)	1109 (54.2%)	1214 (55.1%)	685 (50.9%)	769 (53.5%)	817 (53.5%)	362 (49.0%)	212 (47.9%)	
1 to 3 days	6545 (36.4%)	1722 (36.3%)	866 (34.7%)	766 (37.5%)	803 (36.5%)	515 (38.3%)	540 (37.6%)	554 (36.3%)	302 (40.9%)	171 (38.6%)	

4 to 7 days	994 (5.5%)	276 (5.8%)	170 (6.8%)	111 (5.4%)	129 (5.9%)	85 (6.3%)	82 (5.7%)	106 (6.9%)	46 (6.2%)	36 (8.1%)	
8+ days	476 (2.6%)	140 (3.0%)	89 (3.6%)	59 (2.9%)	56 (2.5%)	60 (4.5%)	46 (3.2%)	49 (3.2%)	29 (3.9%)	24 (5.4%)	

IMD = Index of Multiple Deprivation, OOH = out of hours, GP = general practitioner, GI = gastrointestinal, CNS = central nervous system, ENT = ear nose and throat, PEWS = Paediatric Early Warning Score

Data based on 206,918 observations

Figure S6: Generalised Additive Model showing the relationship between deprivation and risk of admission

Figure S6 describes the relationship between deprivation score (as a continuous measure) and the risk of admission, using a GAM. The relationship is somewhat linear, showing children from the more deprived areas have a smaller likelihood of admission than those from the least deprived. The scaled risk of around 0.4 suggests that there is approximately 50% ($e^{0.4}$)higher unadjusted risk of admission for the most affluent children compared to the most deprived children, across the deprivation scale. Further, the nature of this relationship displayed using the GAM, provides justification to treat IMD as a continuous variable within logistic regression, rather than a grouped variable.

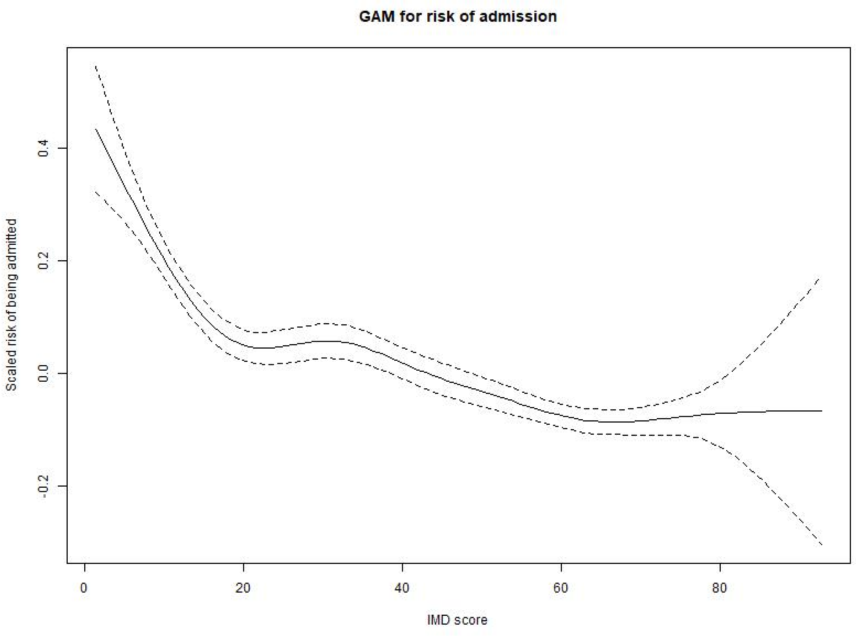

Figure S7: Logit of the predicted probabilities against the continuous IMD score

Several important assumptions of binary multiple linear regression are assessed here. The dependent variable in the model was admission, where a child was either admitted or they weren't (dichotomous). There were one or more independent variables. These were continuous and categorical variables. The assumption of independence of observations cannot be completely satisfied as some patients attended the ED multiple times over the study period. Sandwich estimators attempted to mitigate this. Any continuous independent variables must have a linear relationship with the logit transformation of the dependent variable. This assumption is visually satisfied below by plotting the logit of the predicted probabilities against the continuous variable, after fitting an unadjusted logistic regression model.

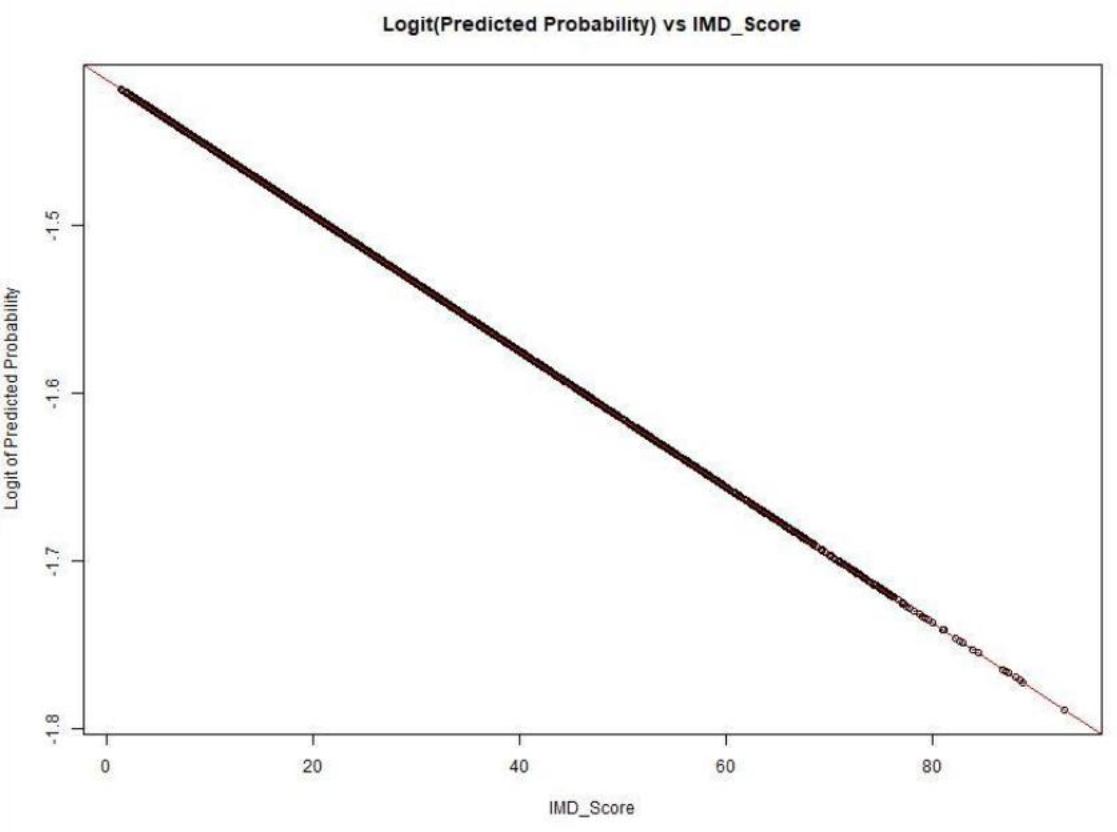

Table S3: Binary logistic model 1: Unadjusted

The following three tables display the binary logistic regression estimates used to derive the odds of admission for Model 1, Model 2 and Model 3 presented in the main results, respectively, using logistic regression IMD as a continuous variable ranges from scores between approximately 0 and 80. For example, Model 1 presents the unadjusted association between odds of admission and deprivation. Results show and odds ratio of 0.996 ($p<0.05$), which is equivalent to approximately 70% increased likelihood for more affluent children to be admitted from the ED, than their most deprived counterparts (0.996^{80}). In other words, children from the most deprived areas were 30% less likely (1.003^{80}) to be admitted than their most affluent counterparts.

Model	Estimate	St.Err	z-value	P-value	Sig
Intercept	-1.413	0.014	-104.10	<0.05	***
IMD	-0.004	0.000	-14.56	<0.05	***

OR = Odds Ratio, CI = Confidence Interval, IMD = Index of Multiple Deprivation
Data based on 206,918 observations.
Residual deviance: 187738 on 206916 degrees of freedom, AIC: 187742

Table S4: Binary logistic model 2: Confounder adjusted

Model 2 describes the odds of admission across the IMD scale, adjusted for potential confounders (age, gender, season and arrival year), and provides the basis for assessing mediation (proxy measures for disease severity). The unadjusted association does not change on adjustment for confounders.

Model	Estimate	St.Err	z-value	P-value	Sig
Intercept	-1.423	0.023	-62.841	<0.05	***
IMD	-0.004	0.000	-15.816	<0.05	***
Arrival age	-0.048	0.001	-36.394	<0.05	***
Gender (Male)	0.021	0.012	1.803	0.072	*
2017 arrival	0.002	0.018	0.125	0.899	
2018 arrival	0.408	0.017	23.939	<0.05	***
2019 arrival	0.368	0.017	21.690	<0.05	***
Summer arrival	0.001	0.017	0.053	0.958	
Autumn arrival	0.079	0.016	4.820	<0.05	***
Winter arrival	0.065	0.017	3.917	<0.05	***

OR = Odds Ratio, CI = Confidence Interval, IMD = Index of Multiple Deprivation
Data based on 206,918 observations.
Residual deviance: 28847 on 206893 degrees of freedom, AIC: 28897

Table S5: Binary logistic model 3: Adjusted for confounders and disease severity measures

Model 3 presents the odds of admission across the IMD scale, additionally adjusted for potential mediators (as described in the DAG, Chapter 3). After additionally adjusting for mediators, there is an observed opposite association between odds of admission and deprivation, resulting in children from the most disadvantaged areas experiencing a 20% increased likelihood of admission, than their most affluent counterparts.

Model	Estimate	St.Err	z-value	P-value	Sig
Intercept	-5.866	0.197	-29.688	<0.05	***
IMD	0.002	0.001	2.042	<0.05	**
Age	-0.053	0.004	-13.944	<0.05	***
Gender(Male)	-0.116	0.036	-3.257	<0.05	**
Year 2017	0.027	0.054	0.505	0.61	
Year 2018	0.189	0.051	3.726	<0.05	***
Year 2019	0.197	0.050	3.910	<0.05	***
Summer	0.641	0.051	1.260	0.21	
Autumn	0.040	0.049	0.820	0.41	
Winter	0.231	0.050	4.644	<0.05	***
Respiratory conditions	0.398	0.063	6.290	<0.05	***
Urological conditions	-0.273	0.104	-2.623	<0.05	**
Gastrointestinal conditions	0.135	0.071	1.901	0.057	*
ENT conditions	-0.874	0.114	-7.667	<0.05	***
Central nervous system	0.474	0.146	3.216	<0.05	**
Other	-0.488	0.057	-8.614	<0.05	***
Triage	0.568	0.024	23.421	<0.05	***
Primary care	-0.872	0.202	-4.103	<0.05	***
OOH	-1.086	0.194	-5.605	<0.05	***
Self	-0.668	0.172	-3.881	<0.05	***
Alder Hey	6.670	0.170	39.235	<0.05	***
PEW score	-2.228	0.315	-7.079	<0.05	***
Number of investigations	0.394	0.019	20.780	<0.05	***
OOH arrival	0.452	0.037	12.162	<0.05	***
Time in ED	0.049	0.013	3.798	<0.05	***

OR = Odds Ratio, CI = Confidence Interval, IMD = Index of Multiple Deprivation
Data based on 206,918 observations
*** p<0.01, ** p<0.05, * p<0.1

Residual deviance: 28847 on 206893 degrees of freedom, AIC: 28897

Table S6: Univariate association between deprivation and important covariates

To further examine the relationship between deprivation and potential risk factors of admission, table S3 presents univariate associations between deprivation and other important variables in this analysis, across all attendances, and all admissions respectively.

	Attendances (n=206,918)	Admissions (n=34,952)
Variable	P value	P value
Triage priority	<0.05	<0.05
Investigation count	<0.05	<0.05
PEWS	0.88	0.37
Diagnosis group	<0.05	<0.05
Referral	<0.05	<0.05
Out of hours	<0.05	0.11
Season	0.29	<0.05
Year	<0.05	0.41
Gender	<0.05	<0.05
Age	<0.05	<0.05
Time in ED	<0.05	<0.05
Inpatient stay	<0.05	<0.05

PEWS = Paediatric Early Warning Score, ED = Emergency Department
Data based on 206,918 observations

Figure S8: Proportion of diagnosis category for all attendances, by deprivation

The following figures present the unadjusted proportion of potential risk factors of admission by deprivation. For this analysis, we use IMD deciles for clearer visual representation and to easily compare the most and least disadvantaged groups in the study population.

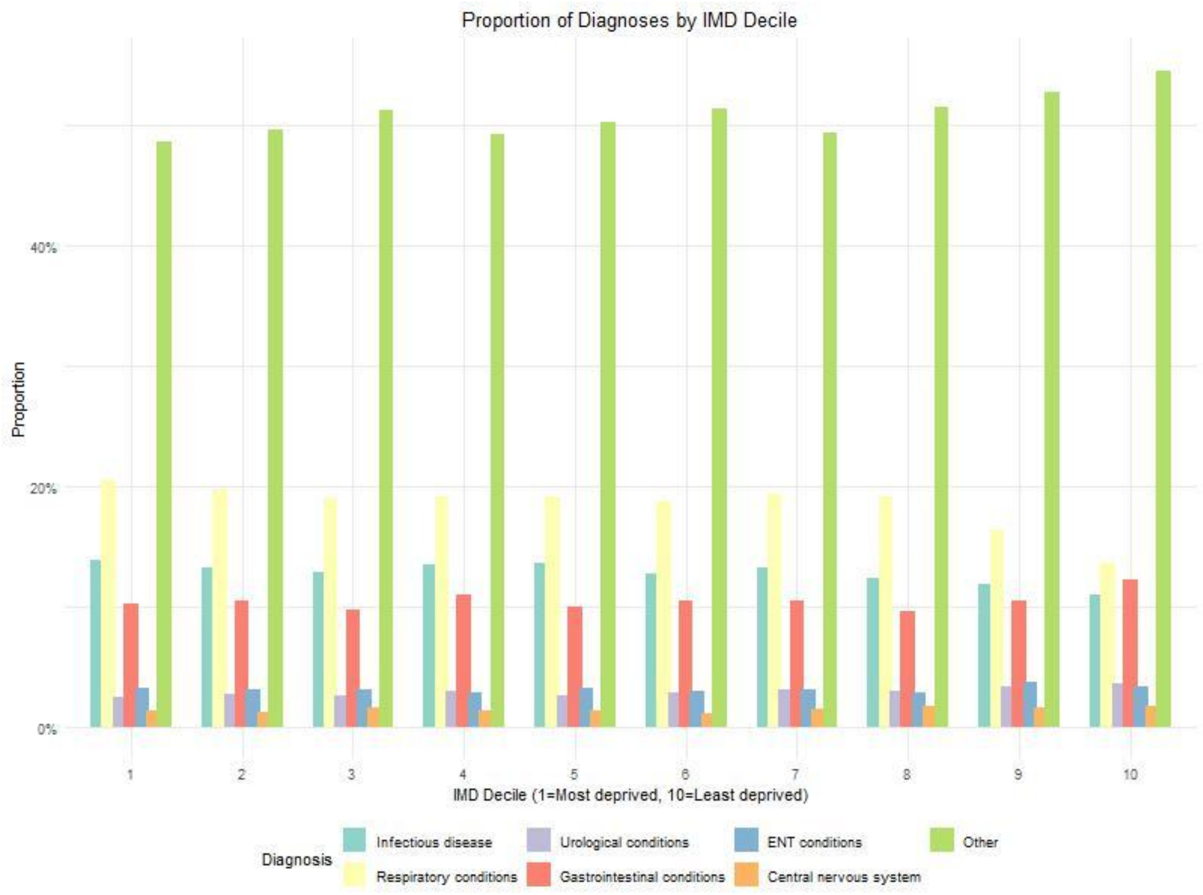

Figure S9: Proportion of diagnosis category for all admissions, by deprivation

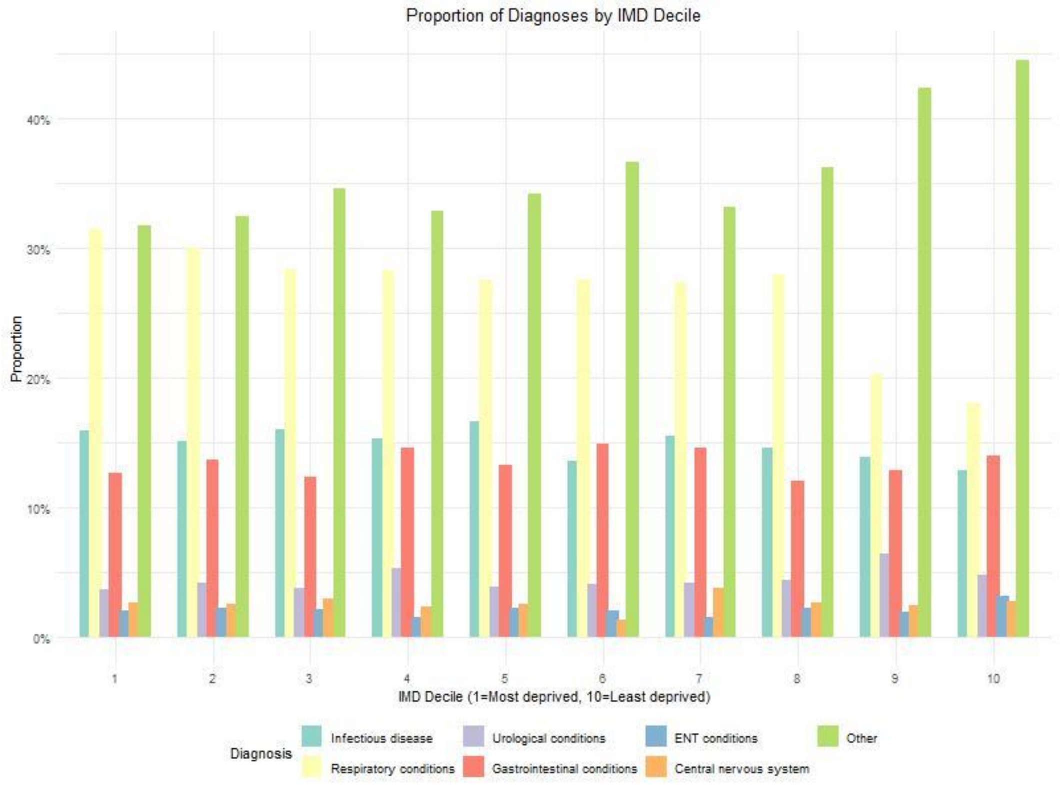

Figure S10: Proportion of referral category for all attendances, by deprivation

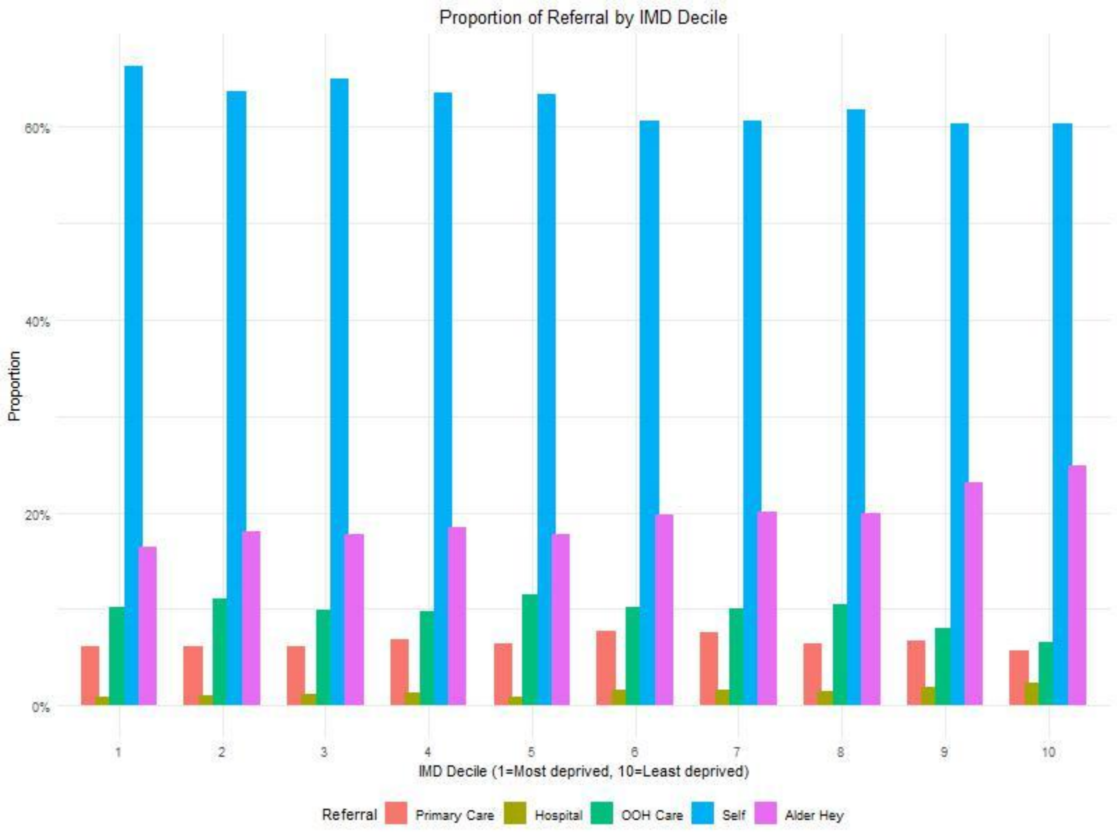

Figure S11: Proportion of referral category for all admissions, by deprivation

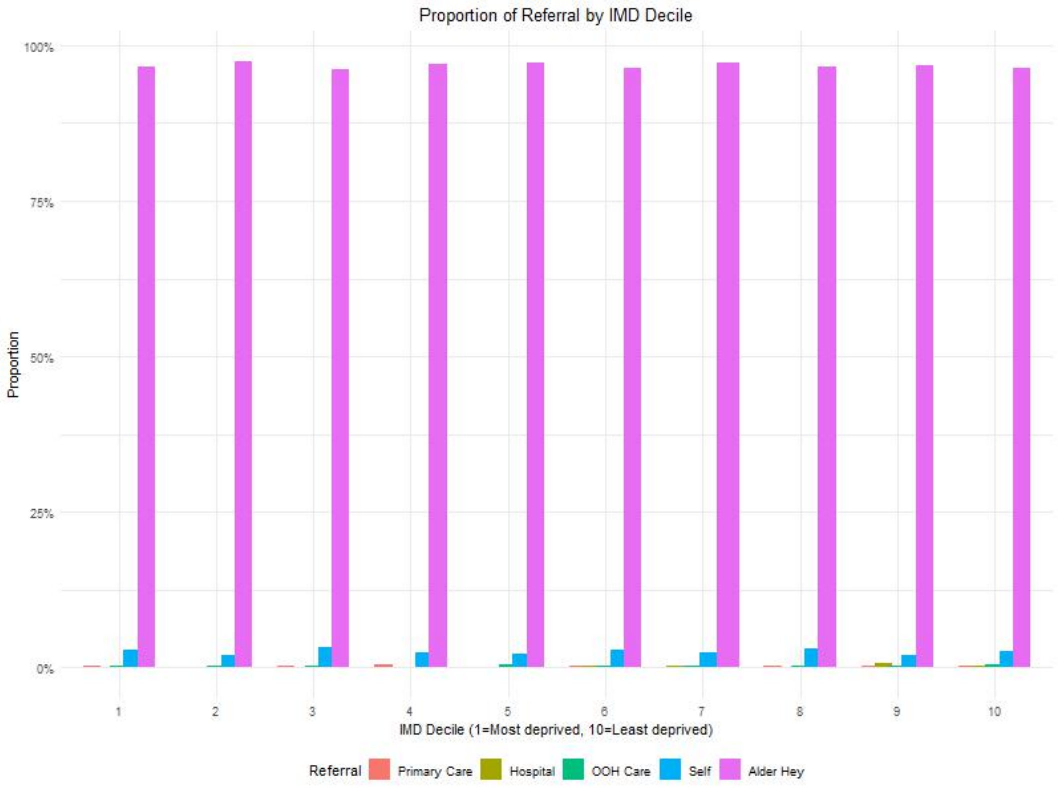

Figure S12: Proportion of number of investigations performed for all attendances, by deprivation

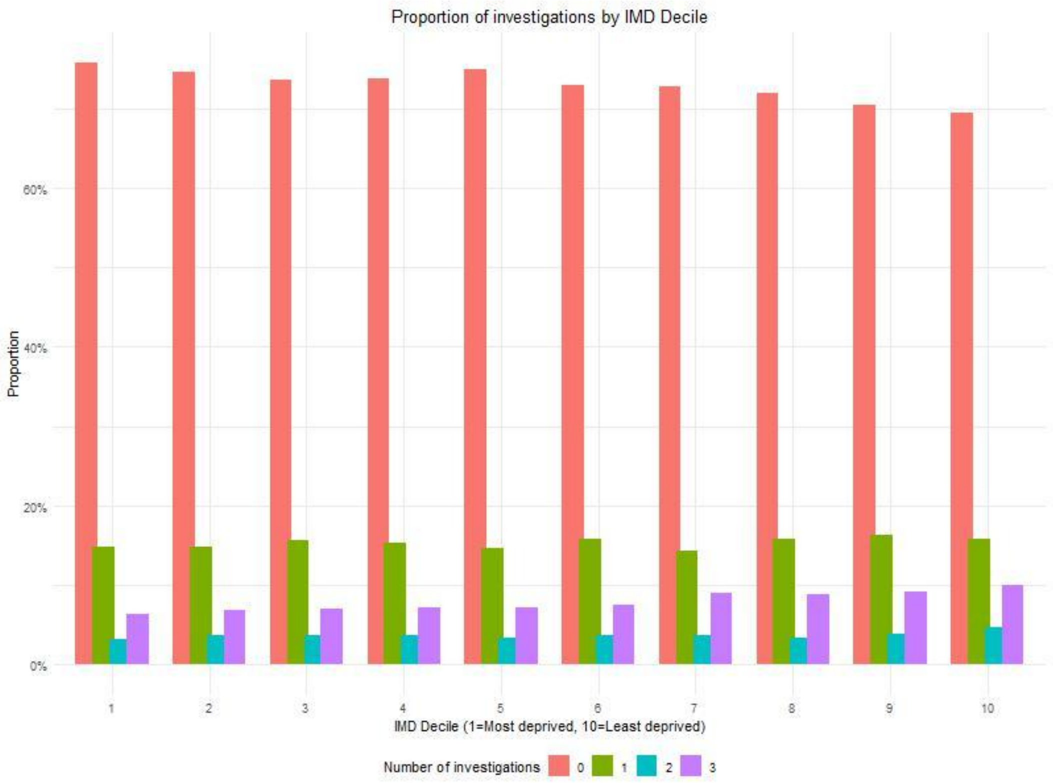

Figure S13: Proportion of number of investigations performed for all admissions, by deprivation

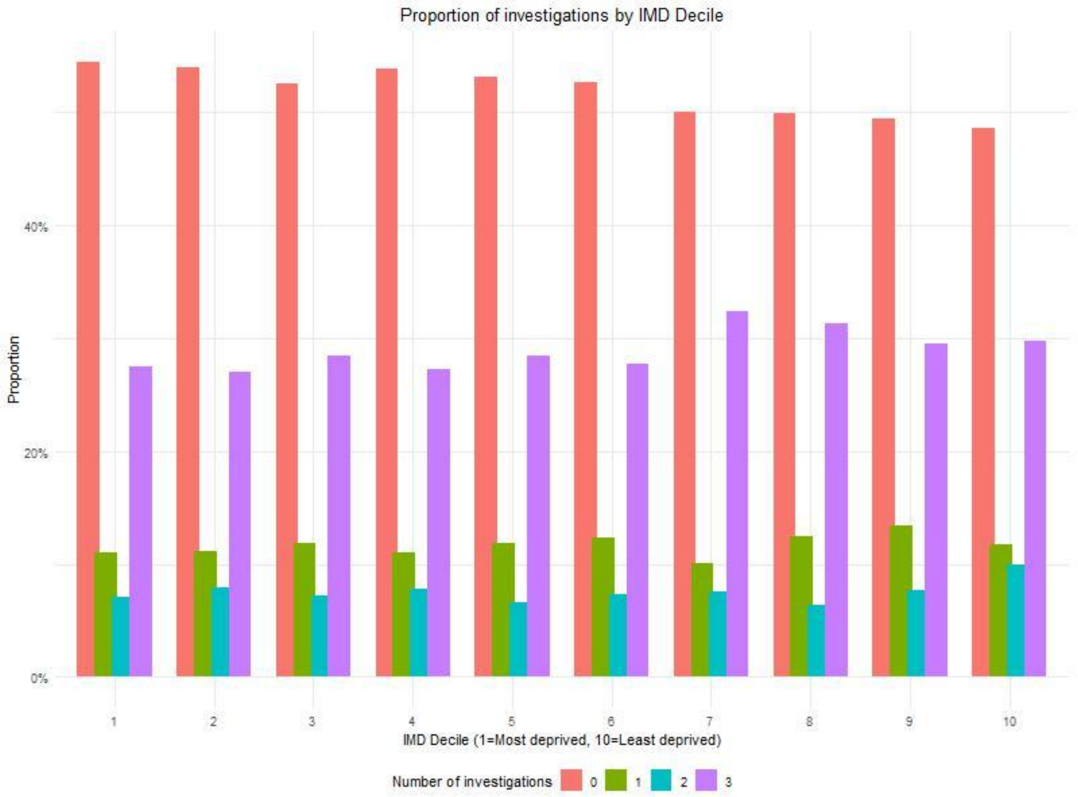

Figure S14: Proportion of out of hours attendance for all attendances, by deprivation

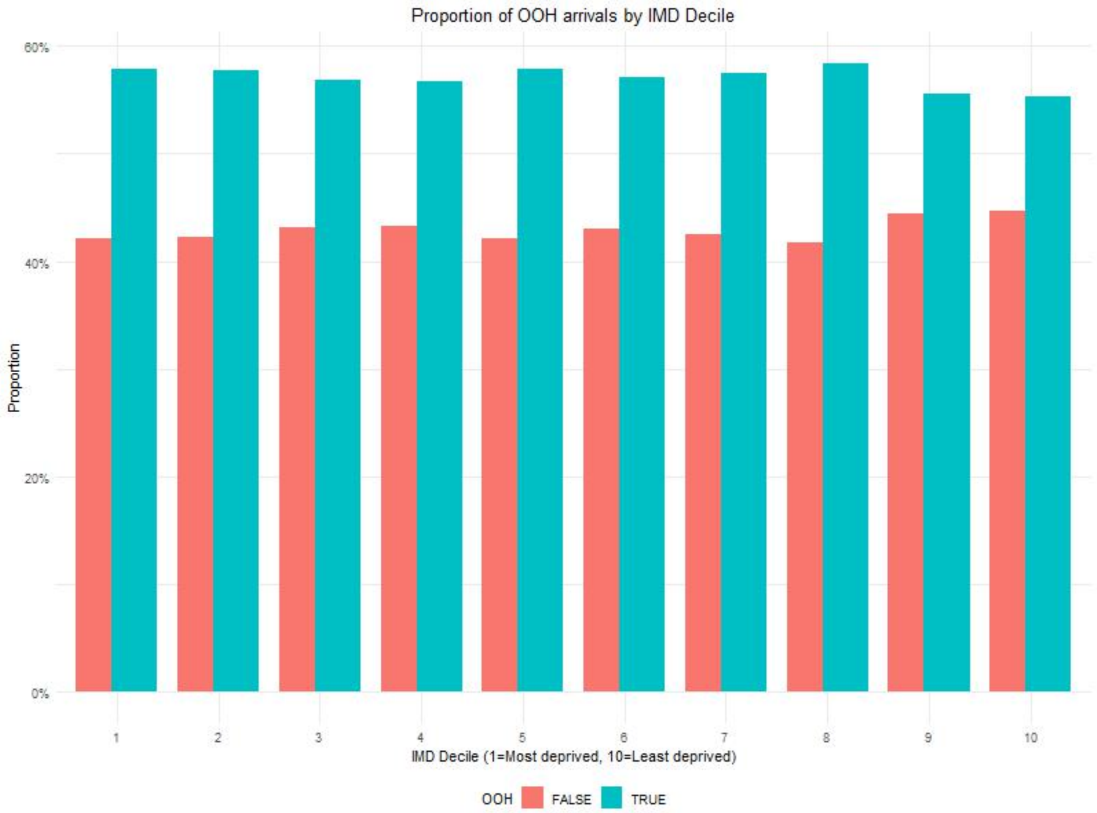

Figure S15: Proportion of out of hours attendance for all admissions, by deprivation

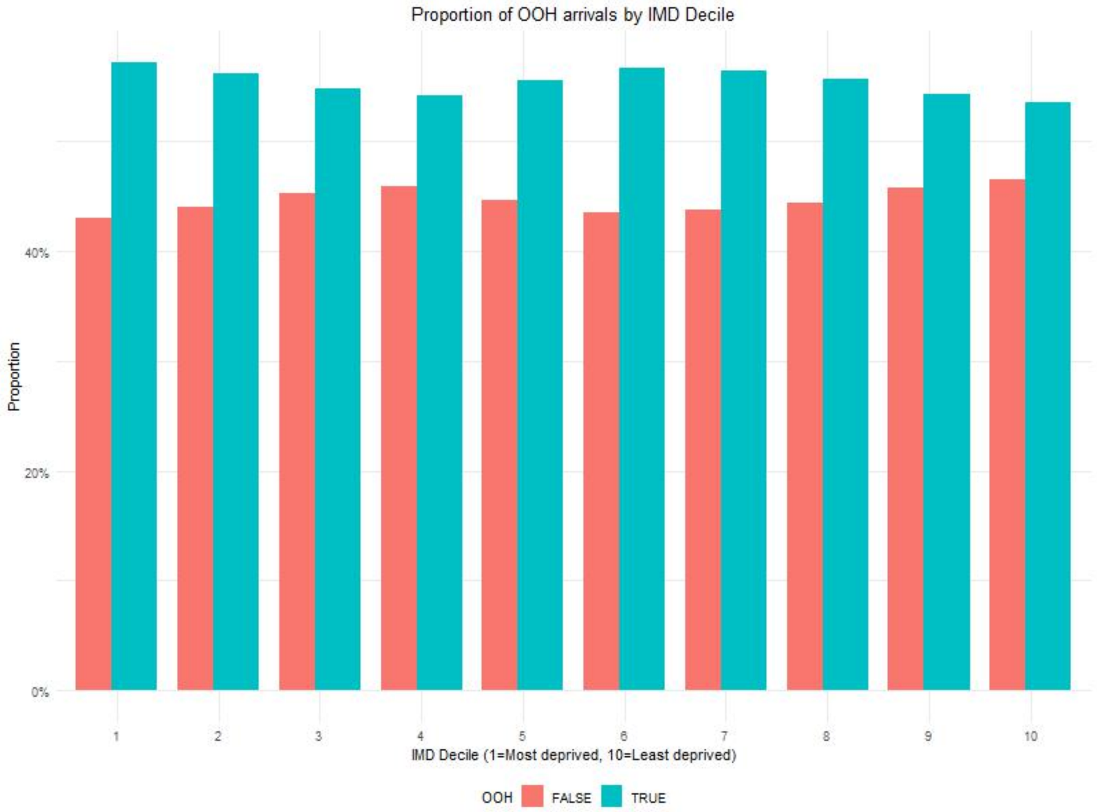

Supplement: online supplemental file 1 [file bmjph-4-2-s001.pdf]
